# Integrating multi-omics data through deep learning with applications to autism risk gene prioritization

**DOI:** 10.1093/bib/bbag588

**Published:** 2026-10-26

**Authors:** Shengtong Han, Di Wang, Wenhui Sheng, Nathanael Chu

**Affiliations:** Department of Community Dental Sciences, School of Dentistry, Marquette University, 1801 West Wisconsin Avenue, 53233 WI, United States; Foxconn, Milwaukee, WI, United States; Department of Mathematical and Statistical Sciences, Marquette University, 1313 West Wisconsin Avenue, 53233 WI, United States; Department of Computer Science, Liberty University, 1971 University Boulevard, 24515 VA, United States

**Keywords:** autism spectrum disorder, ASD risk gene prioritization, multi-omics integration, positive-unlabeled learning, interpretable deep learning, protein–protein interaction networks

## Abstract

Autism spectrum disorder (ASD) is a highly heritable neurodevelopmental disorder. Advances in genetic research, particularly whole-exome sequencing studies, have identified an increasing number of ASD risk genes, especially through large-effect rare variant analyses. However, current rare-variant studies still have limited power to recover the full spectrum of ASD risk genes, leaving a substantial gap between known genes and the estimated genetic architecture of ASD. To address this challenge, we developed Deep-MOTIFs (Multi-Omics inTegratIon For autiSm), a Bayesian-guided positive-unlabeled deep learning framework for multi-omics integration that combines genetic, transcriptomic, and protein-interaction evidence for genome-wide ASD risk-gene prioritization. Deep-MOTIFs achieved stronger performance than existing methods across held-out ASD gene prediction, top-ranked prioritization, positive-label efficiency, and label-noise robustness analyses. Functional enrichment and genome-wide decile analyses further supported the biological relevance of the top-ranked candidates, showing convergence on ASD-related phenotypic annotations and synaptic, neurodevelopmental, and chromatin-regulatory mechanisms. Together, these results support Deep-MOTIFs as a robust framework for ASD risk-gene prioritization under incomplete supervision. Source code is available at: https://github.com/shan-team/Deep-MOTIFs.

## Background and introduction

Autism spectrum disorder (ASD), characterized by deficits in social communication and interaction, as well as restricted or repetitive behaviors and interests, is an early-onset, highly heritable, and heterogeneous neurodevelopmental disorder. It affects *∼ *1 in 36 children in the United States [1], with a male-to-female prevalence ratio of 4:1 [2]. Currently, there is no medication capable of curing ASD, primarily due to its unclear etiology. Over the past two decades, significant progress has been made in understanding the genetic architecture of ASD. For example, studies have shown an 88% concordance rate among monozygotic twins [3], and the heritability of ASD has been estimated to be as high as 90% based on family and twin studies [4]. A range of genetic factors contribute to ASD risk, including common variants that may account for the majority of genetic risk [5, 6], rare variants—particularly rare de novo mutations [7–17] and rare inherited variants [17–21]—as well as copy number variations [22–24]. As a result of these discoveries, the number of high-confidence autism risk genes has increased from a few dozen to nearly 200 [15], with more novel autism risk genes emerging as additional samples are sequenced. Despite rapid progress from large-scale sequencing studies, the number of high-confidence ASD risk genes remains far below the estimated genetic architecture of the disorder: fewer than a few hundred genes have strong evidence, whereas ¿1000 genes may contribute to ASD risk [11, 20, 25]. Closing this gap requires methods that can prioritize genome-wide candidate genes under sparse, heterogeneous, and incomplete genetic evidence, while also determining how multi-omics information can recover risk signals that are not apparent from genetic data alone.

Existing computational approaches address this gap from complementary but incomplete angles, including models based on genetic evidence [26], human brain gene-level features [27], and multi-omics integration across genetic, transcriptomic, and protein-interaction data. For example, TADA [25] was introduced to integrate different variant types, such as inherited variants and *de novo* mutations, through a Bayesian approach that only focused on genetic data and is widely used in autism studies [12–16]. But they are driven mainly by genetic association signals and do not directly integrate developmental expression or protein-interaction context. Liu *et al*. [28, 29] proposed a network-based approach, DAWN, to integrate gene co-expression network and protein–protein interaction (PPI) network, and well applied by the studies [30–33]. But DAWN suffers from several limitations: (i) the potential risk genes need to be co-expressed with other risk genes (ii) it is not scalable to nearly 20 000 genes genome-wide, due to the small sample size in brain gene expression data and high-dimensional parameters in the network model. DeepND [34] extended this idea by learning disease–gene representations from network topology and gene-level features, demonstrating the value of deep learning for network-based gene prioritization. In contrast, forecASD [35] broadened the evidence base by integrating genetic, transcriptome, and protein-interaction information at genome scale, showing that multi-omics data can improve ASD risk-gene prioritization. Yet two questions remain unresolved: whether multi-omics information has been fully utilized beyond tree-based ensemble learning, and whether the standard positive–negative training formulation is appropriate for ASD gene discovery. Curated ASD genes provide reliable positive labels, whereas available negative labels are better viewed as weak reference negatives rather than confirmed non-risk genes; the remaining genome-wide genes form an unlabeled mixture that may include undiscovered ASD risk genes.

Here, we address these challenges with Deep-MOTIFs (Multi-Omics inTegratIon For autiSm), a Bayesian-guided positive-unlabeled (PU) ranking framework for ASD risk-gene prioritization. Rather than treating available negative labels as confirmed non-risk genes, Deep-MOTIFs uses them as weak reference negatives, together with curated ASD positives, to calibrate a gene-level empirical prior from genetic association evidence, BrainSpan developmental expression, and STRING-derived network features. This empirical prior provides a soft negative responsibility for unlabeled genes. To account for prior uncertainty, Deep-MOTIFs constructs entropy-calibrated lower and upper responsibility weights around this empirical negative responsibility and uses the conservative lower-side weight in both the weighted non-negative PU loss and the pairwise PU ranking loss. A multi-view neural posterior ranker then integrates the same genetic, developmental, and network evidence to learn ASD-risk ranking signals under incomplete supervision. Finally, the empirical prior score and learned PU posterior score are combined by reciprocal-rank fusion to generate the genome-wide ranking of candidate ASD risk genes.

Our contributions are summarized as follows: (i) we formulate ASD risk-gene prioritization as a PU ranking problem under incomplete supervision from curated positives, weak reference negatives, and genome-wide unlabeled candidates; (ii) we develop an empirical-Bayes formulation for PU learning that uses a calibrated ASD-risk prior to define entropy-regularized responsibility weights and establishes an endpoint ordering of the corresponding responsibility-weighted PU risks; building on this formulation, we propose Deep-MOTIFs, a multi-view neural ranker integrating genetic association, developmental transcriptomic, and protein-interaction evidence; and (iii) we show that Deep-MOTIFs outperforms existing ASD-specific, PU, and general-purpose methods in held-out risk-gene prioritization, remains robust under incomplete and noisy supervision, and nominates biologically coherent candidates enriched for ASD-relevant phenotypic, synaptic, neurodevelopmental, and regulatory signals.

## Multi-omics samples and Deep-MOTIFs

### Multi-omics data used in autism study

Figure 1 provides an overview of the multi-omics evidence sources, the resulting gene-level feature construction, and the incomplete-label setting that motivates the Bayesian-guided PU formulation of Deep-MOTIFs. We constructed gene-level multi-omics features from three complementary sources: rare-variant genetic association evidence, developmental brain transcriptome evidence, and protein-interaction network evidence, following the general data organization of forecASD [35] but using updated ASD genetic evidence. For genetic association features, we used gene-level results from the whole exome sequence (WES) study of Fu *et al*. [15], which analyzed aggregated family-trio and case-control samples and provided TADA p-values and FDR values for each gene, and the whole genome sequence (WGS) study of MSSNG reported by Trost *et al*.[16], from which gene-level FDR values were included. Because ASD is a neurodevelopmental disorder, developmental brain expression provides complementary information beyond direct genetic association signals [36]. We used BrainSpan developmental transcriptome data [37], generated from 1340 brain tissues from 57 developing and adult post-mortem brains, which have been widely used in ASD studies to characterize spatiotemporal expression and co-expression patterns [14, 15, 21, 28, 29, 38]. Following the BrainSpan preprocessing strategy used in forecASD [35], brain regions with fewer than 20 samples were removed, expression trajectories within each retained region were smoothed over developmental age and interpolated to 50 common time points, and the resulting gene-level profiles were standardized, yielding a *16 × 50* BrainSpan matrix for each gene. Finally, to incorporate functional network context, we used protein–protein interaction information [39–42], focusing on STRING [43], which has been widely used in ASD-related studies [35, 44, 45]. Following forecASD, STRING interactions with combined scores ¿400 were retained, and multiple protein-level interactions mapping to the same gene pair were collapsed by taking the maximum score as the gene-level edge weight.

**Figure 1:**
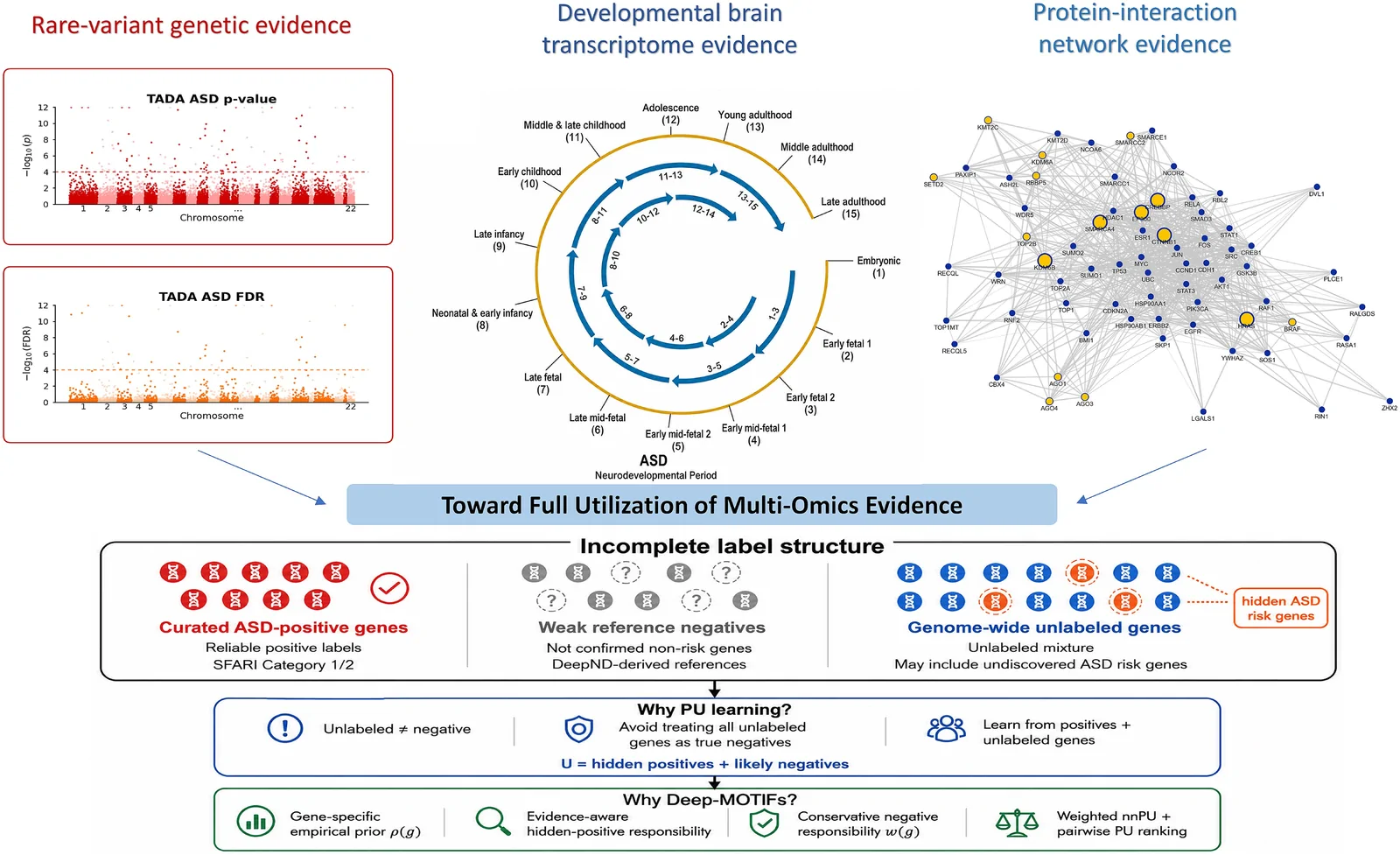
Multi-omics feature construction and incomplete-label motivation of Deep-MOTIFs.

We obtained ASD labels from the updated SFARI Gene database (https://gene.sfari.org/), where genes are classified by evidence level. This release contains 233 Category 1 genes with high-confidence ASD evidence and 708 Category 2 genes with strong candidate evidence. We used genes in both categories 1 and 2 as curated ASD-positive labels. For reference negatives, we followed the label construction used in DeepND [27], retaining 727 DeepND-derived non-mental-health-related genes collected from OMIM and Krishnan *et al*. [46]. These genes were not treated as confirmed non-ASD genes, because the absence of current ASD annotation does not exclude future ASD relevance. Instead, they were used only as weak reference labels for empirical-prior calibration, while the remaining genome-wide genes were treated as unlabeled candidates that may contain undiscovered ASD risk genes. This label structure motivates the Bayesian-guided PU formulation of Deep-MOTIFs: PU learning reduces dependence on reliable negative labels, but its central difficulty is that the latent positive or negative responsibility of each unlabeled gene is unobserved. Deep-MOTIFs addresses this difficulty by estimating a gene-level empirical prior, converting it into conservative entropy-shrunk negative-responsibility weights, and using these weights in both non-negative PU risk and pairwise PU ranking.

### The framework of Deep-MOTIFs

Deep-MOTIFs is designed to address two closely related methodological limitations in genome-wide ASD risk-gene prioritization. First, although multi-omics resources have enabled the joint use of genetic association evidence, developmental brain expression, and protein-interaction networks, these data sources represent distinct biological layers and differ substantially in scale, structure, sparsity, and noise level. Their integration therefore requires a model that can retain modality-specific information while learning cross-modal evidence for gene ranking. Second, the supervision available for ASD gene discovery is intrinsically incomplete. Many ASD gene-prioritization models formulate the task as standard positive–negative classification, using curated ASD genes as positives and randomly sampled background genes, selected non-psychiatric genes, or otherwise unlabeled genes as negatives [27, 35, 47, 48]. Such negative sets are convenient for the training purpose, but they should not be interpreted as biologically verified non-risk genes. SFARI Gene is an evolving database, and new ASD risk genes continue to be added as genetic studies expand; e.g. the Q2 2024 SFARI release included 27 ASD risk genes that were absent from the Q4 2023 release. Thus, genes treated as negatives in one training set may later acquire ASD evidence, making hard positive–negative learning poorly matched to genome-wide ASD gene discovery. Accordingly, Deep-MOTIFs is organized as a three-stage framework, as summarized in Fig. 2: empirical-prior calibration, bound-aware PU posterior learning, and rank-space prior-posterior fusion.

**Figure 2:**
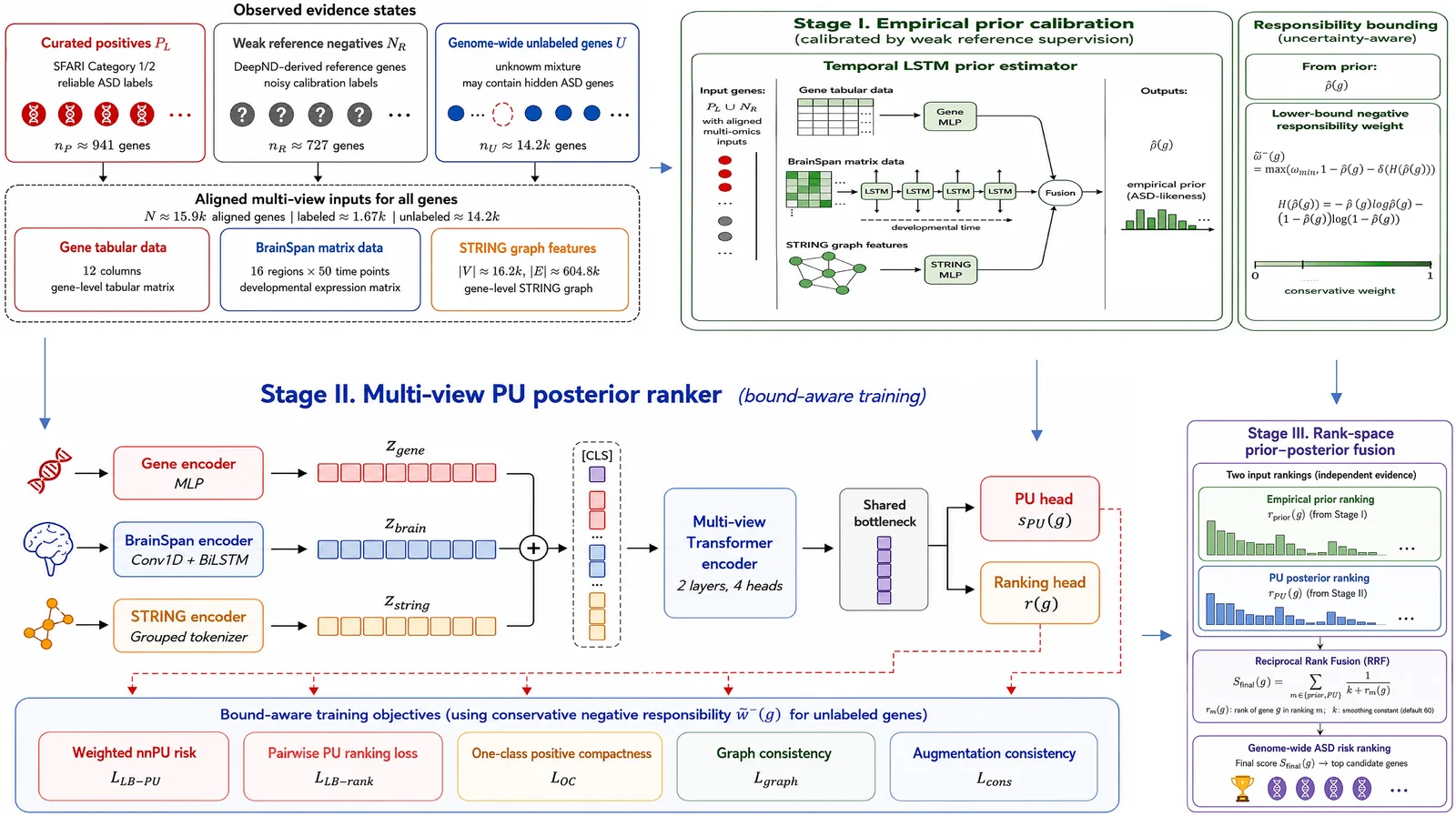
The graphical illustration of Deep-MOTIFs In Stage I, an empirical prior estimator is trained from curated positives and weak reference negatives using aligned multi-view gene representations, and its output is transformed into conservative entropy-shrunk negative-responsibility weights for unlabeled genes. In Stage II, a multi-view PU posterior ranker integrates gene tabular data, BrainSpan developmental expression matrices, and STRING graph features through view-specific encoders and a Transformer fusion module. The posterior ranker is trained with bound-aware objectives weighted by the conservative negative responsibilities. In Stage III, reciprocal-rank fusion combines the empirical prior ranking with the PU posterior ranking, yielding the final genome-wide ASD risk ranking.

Deep-MOTIFs formulates ASD risk-gene discovery as a Bayesian-guided PU ranking problem with multi-omics neural evidence integration. Let $Y_{g} \in \{0,1\}$ denotes the unobserved true ASD-risk status of gene *g*, with $Y_{g}=1$ indicating an ASD risk gene, and let $X_{g}$ denotes its observed multi-omics feature vector. Unlike standard positive–negative classification, which assumes that both positive and negative labels are observed, our setting contains reliable positive labels, weak reference negatives, and a large set of genome-wide genes with unknown $Y_{g}$. We therefore first estimate a gene-level empirical prior and calibrate it to obtain the corresponding hidden-positive responsibility: 


(1) \begin{align*}& \hat{\pi}(g) \approx P(Y_{g}=1\mid X_{g}), \qquad \hat{\rho}(g)=\operatorname{Calib}\{\hat{\pi}(g)\}.\end{align*}

Here, $\hat{\pi }(g)$ represents the empirical ASD-likeness prior of gene *g*, whereas $\hat{\rho }(g)$ denotes its calibrated hidden-positive responsibility. The complement $1-\hat{\rho }(g)$ provides an empirical estimate of the latent negative responsibility of gene *g*. However, using this point estimate directly may over-penalize genes with uncertain prior evidence. We therefore define an entropy-shrunk negative responsibility: 


(2) \begin{align*}& \widetilde{w}^{-}(g) = \max\left\{ w_{\min}, \,1-\hat{\rho}(g)-\delta H(\hat{\rho}(g)) \right\}.\end{align*}

where $H(\hat{\rho }(g))$ denotes the entropy-based uncertainty of the calibrated empirical responsibility, $\delta \geq 0$ controls the strength of uncertainty shrinkage, and $w_{\min }$ prevents the negative-responsibility weight from degenerating to zero. Larger weights are assigned to genes with confidently low ASD responsibility, whereas genes with ambiguous or ASD-like prior evidence contribute more conservatively to PU learning. The entropy definition and the corresponding responsibility-weighted risk ordering are provided in Appendix.

Genetic features are encoded as tabular association evidence, BrainSpan expression is represented as a structured *16 × 50* developmental matrix, and STRING information is encoded through fold-specific network-proximity features. These views are passed into a multi-omics neural ranker that learns posterior evidence for ASD association. The training objective combines entropy-weighted non-negative PU risk, pairwise PU ranking loss, one-class compactness, graph-neighbor consistency, and augmentation consistency. Finally, Deep-MOTIFs combines the empirical prior score and the learned PU posterior score through reciprocal-rank fusion to produce a genome-wide ranking of candidate ASD risk genes. The empirical prior score is calculated by an LSTM estimator trained on positives and weak reference negatives with binary cross-entropy loss. The formal problem definition, model architecture, entropy-calibrated responsibility weighting, risk-ordering formulation, training objective, and algorithmic pseudo-code are provided in Appendix.

## Deep-MOTIFs outperforms competing methods in autism spectrum disorder risk-gene prioritization

We benchmarked Deep-MOTIFs against a comprehensive set of ASD-specific, PU, and general-purpose machine learning baselines for gene prioritization. The ASD-focused comparisons included forecASD [35], the functional relationship network-based random forest (FRN-RF) model of Duda *et al*. [49], DeepND [34], DeepASDPred [50], and the brain-network SVM model of Krishnan *et al*. [46]. For PU-learning comparisons, we included nnPU-MLP [51], VPU-MLP [52], and Dist-PU-MLP [53], representing non-negative risk correction, variational PU learning, and label-distribution alignment, respectively. To further assess performance against strong tabular-learning alternatives, we also included XGBoost [54], DeepGBM [55], TabNet [56], SAINT [57], and FT-Transformer [58]. Together, these baselines cover tree-based ensemble learning, kernel-based classification, functional-network models, graph neural networks, convolutional and recurrent architectures, and Transformer networks. For fairness, all methods were evaluated using the same gene-level multi-omics input and the same positive and weak-reference-negative label construction described above. This comparison provides a solid evaluation of whether Deep-MOTIFs improves ASD risk-gene prioritization beyond all ASD-specific methods, PU approaches and modern generic predictors. Implementation details, hyperparameter settings, and method-specific adaptations to the unified multi-omics feature space are provided in Appendix.

For evaluation, we report complementary classification and prioritization metrics. Accuracy and F1 score assess threshold-based prediction performance, whereas PR-AUC and ROC-AUC measure threshold-independent discrimination. MCC-AUC and MCC-max summarize balanced classification quality across rank-derived decision thresholds. P@10 and P@50 measure the fraction of ASD-positive genes among the top 10 and top 50 held-out labeled genes ranked by out-of-fold scores, respectively; nDCG@50 gives additional credit when held-out positives appear closer to the top; and Lift@50 quantifies top-50 enrichment relative to random ranking. All metrics are oriented so that larger values indicate better performance. We repeated the full experiment across five independent runs, each using an independently sampled balanced reference set. Within each run, we performed stratified five-fold cross-validation, yielding out-of-fold predictions for all labeled genes. Training-fold positives were never counted as hits for the corresponding fold-level ranking metrics. For Deep-MOTIFs, the empirical prior estimator, PU ranker, and final rank-space fusion were trained strictly within the training folds to avoid information leakage. Genome-wide novel-candidate analyses were performed separately on unlabeled genes after excluding all labeled positives and weak-reference negatives.

### Benchmarking Deep-MOTIFs by cross validations

As shown in Table 1, Deep-MOTIFs achieved the strongest overall performance among ASD-specific, PU, and general-purpose baselines. Relative to forecASD, Deep-MOTIFs improved F1, PR-AUC, and ROC-AUC by 9.2%, 9.0%, and 5.8%, respectively. Compared with DeepND, Deep-MOTIFs achieved gains of 9.2% in F1, 7.9% in PR-AUC, and 8.4% in ROC-AUC. Among the PU-learning baselines, Dist-PU-MLP performed best, with PR-AUC of 0.8210 and ROC-AUC of 0.7829; Deep-MOTIFs achieved higher values on both metrics, with PR-AUC of 0.8624 and ROC-AUC of 0.8339, as well as higher F1 and MCC-AUC. These comparisons indicate that the gain of Deep-MOTIFs is not simply due to replacing positive–negative classification with a generic PU objective, but to combining PU learning with empirical-prior calibration, conservative responsibility weighting, and rank-space prior–posterior fusion. The advantage of Deep-MOTIFs was also evident in ranking-oriented metrics, which are particularly important for genome-wide risk-gene prioritization. Relative to forecASD, Deep-MOTIFs increased P@50 from 0.9000 to 0.9768, nDCG@50 from 0.9183 to 0.9825, and Lift@50 from 1.8000 to 1.9536. It also improved MCC-AUC from 0.3428 to 0.3925 and MCC-max from 0.4697 to 0.5701, indicating stronger balanced classification quality across rank-derived thresholds. These results show that Deep-MOTIFs not only improves prediction on labeled genes, but also produces a more reliable ordering of high-priority candidates for downstream biological validation.

**Table 1:** Held-out ASD risk-gene prioritization performance. Deep-MOTIFs was evaluated against ASD-specific, P, and general-purpose machine-learning baselines using the same updated multi-omics feature space. Each method was evaluated over five independent repetitions, and each repetition used five-fold cross-validation. Values denote mean*± *standard deviation across the five repetitions. Higher values indicate better performance for all metrics

***ASD-specific gene-prioritization baselines***										
**Method**	**Classification**				**Top-ranked prioritization**			**MCC- and enrichment-based ranking**		
	**Accuracy**	**F1**	**PR-AUC**	**ROC-AUC**	**P@10**	**P@50**	**nDCG@50**	**MCC-AUC**	**Lift@50**	**MCC-max**
**Deep-MOTIFs**	**0.7585*± *0.0052**	**0.7564*± *0.0037**	**0.8624*± *0.0017**	**0.8339*± *0.0030**	**1.0000*± *0.0000**	**0.9768*± *0.0066**	**0.9825*± *0.0046**	**0.3925*± *0.0040**	**1.9536*± *0.0131**	**0.5701*± *0.0098**
forecASD	0.7020*± *0.0104	0.6927*± *0.0093	0.7913*± *0.0065	0.7882*± *0.0040	0.9800*± *0.0346	0.9000*± *0.0123	0.9183*± *0.0129	0.3428*± *0.0048	1.8000*± *0.0246	0.4697*± *0.0085
FRN-RF	0.7399*± *0.0073	0.7292*± *0.0071	0.8266*± *0.0042	0.8057*± *0.0050	0.9920*± *0.0110	0.9448*± *0.0052	0.9570*± *0.0053	0.3627*± *0.0056	1.8896*± *0.0104	0.5145*± *0.0132
DeepND	0.7058*± *0.0096	0.6926*± *0.0062	0.7991*± *0.0019	0.7691*± *0.0045	0.9800*± *0.0245	0.9408*± *0.0153	0.9529*± *0.0106	0.3194*± *0.0047	1.8816*± *0.0307	0.4642*± *0.0026
DeepASDPred	0.6094*± *0.0268	0.5433*± *0.0525	0.7017*± *0.0209	0.6982*± *0.0142	0.8840*± *0.0537	0.8024*± *0.0241	0.8242*± *0.0291	0.2361*± *0.0169	1.6048*± *0.0482	0.3383*± *0.0155
Krishnan-SVM	0.6682*± *0.0072	0.6487*± *0.0199	0.7142*± *0.0125	0.7313*± *0.0091	0.9840*± *0.0261	0.9112*± *0.0175	0.9279*± *0.0129	0.2625*± *0.0170	1.8224*± *0.0351	0.3769*± *0.0126
***PU learning baselines***										
**Method**	**Accuracy**	**F1**	**PR-AUC**	**ROC-AUC**	**P@10**	**P@50**	**nDCG@50**	**MCC-AUC**	**Lift@50**	**MCC-max**
Dist-PU-MLP	0.6702*± *0.0183	0.7021*± *0.0050	0.8210*± *0.0021	0.7829*± *0.0043	**1.0000*± *0.0000**	0.9760*± *0.0080	0.9822*± *0.0056	0.3376*± *0.0050	1.9520*± *0.0160	0.4858*± *0.0152
nnPU-MLP	0.5131*± *0.0057	0.6540*± *0.0078	0.6631*± *0.0184	0.6068*± *0.0245	**1.0000*± *0.0000**	0.8013*± *0.0303	0.8495*± *0.0220	0.1310*± *0.0254	1.6027*± *0.0606	0.2768*± *0.0145
VPU-MLP	0.4970*± *0.0023	0.6574*± *0.0022	0.5516*± *0.0108	0.5256*± *0.0034	0.6667*± *0.0611	0.5893*± *0.0439	0.6184*± *0.0529	0.0311*± *0.0043	1.1786*± *0.0876	0.1458*± *0.0090
***General-purpose machine-learning and deep-learning baselines***										
**Method**	**Accuracy**	**F1**	**PR-AUC**	**ROC-AUC**	**P@10**	**P@50**	**nDCG@50**	**MCC-AUC**	**Lift@50**	**MCC-max**
DeepGBM	0.7496*± *0.0069	0.7377*± *0.0098	0.8487*± *0.0041	0.8229*± *0.0040	**1.0000*± *0.0000**	0.9696*± *0.0061	0.9770*± *0.0043	0.3810*± *0.0045	1.9392*± *0.0121	0.5339*± *0.0081
XGBoost	0.7451*± *0.0094	0.7341*± *0.0102	0.8436*± *0.0088	0.8190*± *0.0082	0.9960*± *0.0089	0.9632*± *0.0115	0.9722*± *0.0101	0.3778*± *0.0095	1.9264*± *0.0229	0.5317*± *0.0100
SAINT	0.7413*± *0.0013	0.7279*± *0.0073	0.8361*± *0.0006	0.8083*± *0.0044	0.9880*± *0.0110	0.9648*± *0.0033	0.9702*± *0.0046	0.3631*± *0.0055	1.9296*± *0.0067	0.5267*± *0.0065
FT-Transformer	0.7355*± *0.0088	0.7033*± *0.0088	0.8342*± *0.0044	0.8016*± *0.0045	**1.0000*± *0.0000**	0.9712*± *0.0111	0.9792*± *0.0083	0.3573*± *0.0048	1.9424*± *0.0222	0.5102*± *0.0116
TabNet	0.6852*± *0.0106	0.6825*± *0.0109	0.7642*± *0.0096	0.7477*± *0.0094	0.9200*± *0.0316	0.8904*± *0.0146	0.9012*± *0.0175	0.2932*± *0.0112	1.7809*± *0.0292	0.4217*± *0.0169

Bold values are highest value of all methods.

Figure 3 compares the methods across rank thresholds and precision–recall trade-offs. The MCC rank-threshold curve evaluates the quality of candidate lists obtained by selecting the top-ranked *x%* of genes, reflecting how robust a method is to different downstream validation budgets. Deep-MOTIFs dominates competing methods over a broad range of rank thresholds, showing that its prioritization remains reliable not only for the very top candidates but also when the candidate list is expanded. Deep-MOTIFs preserves higher precision across most recall levels, indicating that increased recovery of ASD-positive genes does not require a disproportionate increase in false positives. These curve-level results support the aggregate performance in Table 1 and suggest that Deep-MOTIFs yields a more usable genome-wide ranking for practical ASD risk-gene discovery.

**Figure 3:**
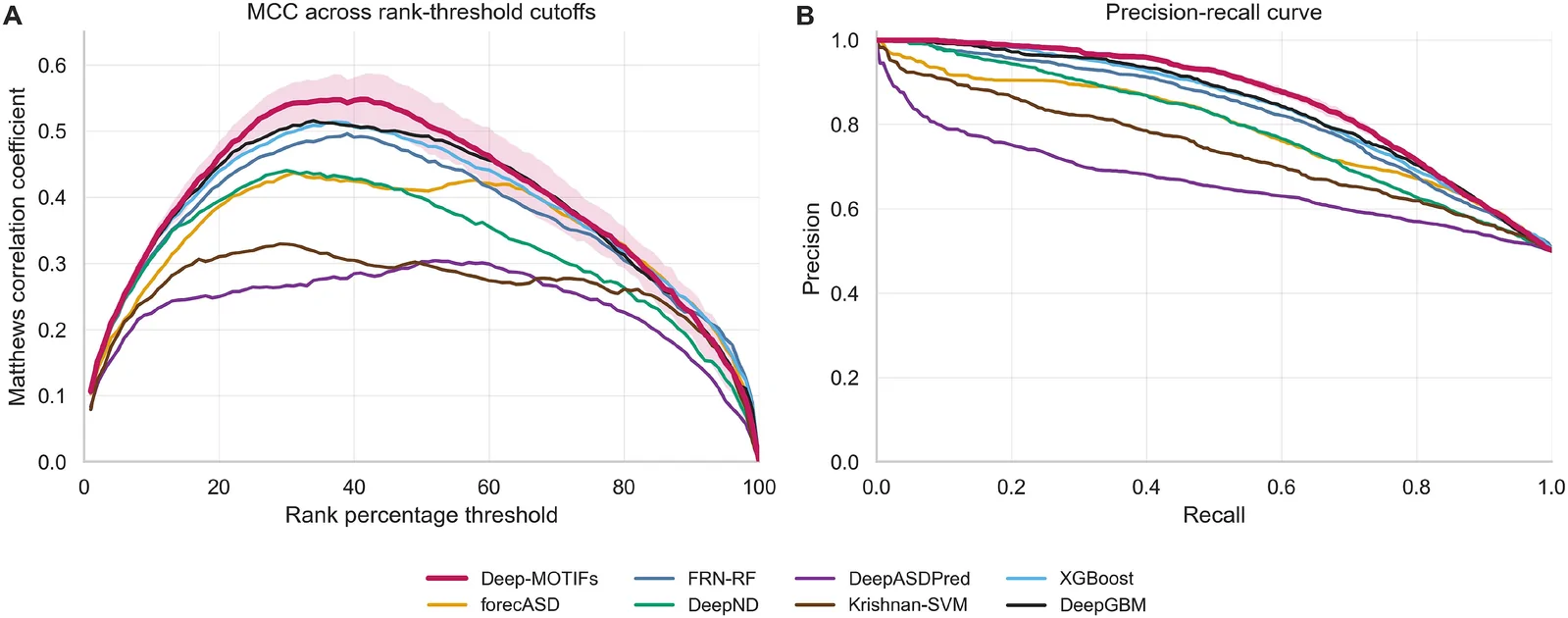
Rank-threshold and precision–recall behavior of Deep-MOTIFs and competing methods. (A) MCC across rank-percentage thresholds, evaluating how well each method converts a genome-wide ranking into candidate gene lists of varying sizes. (B) Precision–recall curves computed from held-out fold predictions, evaluating performance across recall levels. Curves show mean performance across five independent repetitions of five-fold cross-validation, with the shaded band indicating one standard deviation for Deep-MOTIFs.

### Positive-label efficiency under incomplete autism spectrum disorder annotations

To evaluate whether each method remains effective when ASD-positive annotations are incomplete, we progressively reduced the number of positive genes used for training while evaluating all models on the same held-out genes. This analysis addresses a central difficulty in ASD risk-gene prioritization: known ASD genes provide reliable positive evidence, but the current positive set is incomplete and continues to expand as new genetic evidence accumulates. Figure 4 shows the qualitative behavior of each method as positive supervision decreases. Deep-MOTIFs exhibits a stable performance profile across supervision levels, particularly for PR-AUC and ROC-AUC, indicating that its ability to rank ASD-positive genes is only mildly affected by reduced positive-label availability.

**Figure 4:**
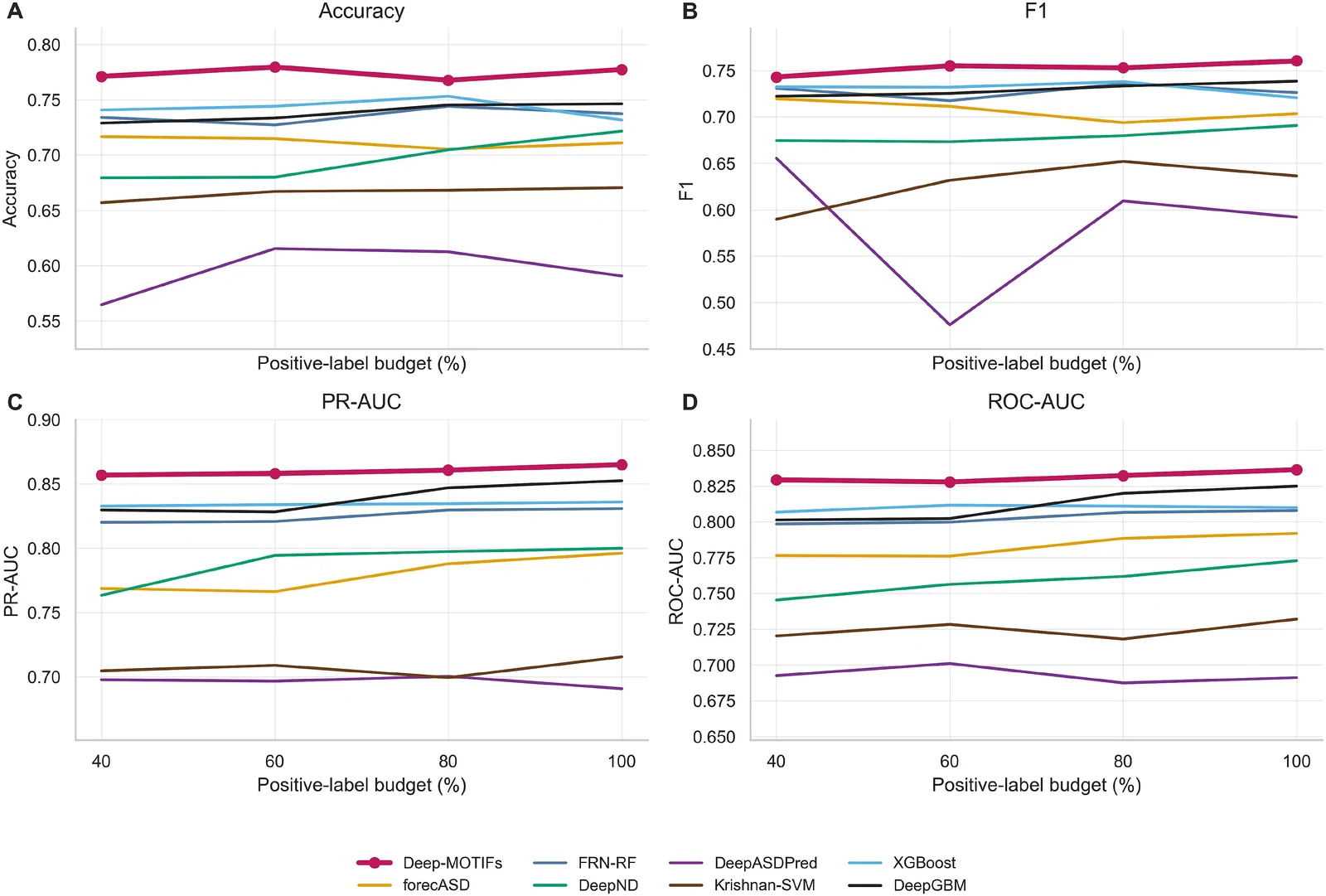
Robustness to limited positive-label supervision across different methods. We evaluated Deep-MOTIFs and competing methods under progressively reduced ASD-positive label budgets, retaining 40%, 60%, 80%, or 100% of the available positive labels for model training while keeping the evaluation protocol fixed. (A–D), Accuracy, F1, PR-AUC, and ROC-AUC as functions of the positive-label budget. Deep-MOTIFs remains consistently competitive or superior across all supervision levels, suggesting that the Bayesian-guided PU ranking formulation can extract stable multi-omics risk signals even when only a limited fraction of known ASD genes is available for training.

Table 2 provides the corresponding quantitative summary across the four supervision levels. Deep-MOTIFs achieved the highest mean performance in all four metrics. Relative to forecASD, Deep-MOTIFs improved F1 by 6.5%, PR-AUC by 10.3%, and ROC-AUC by 6.2%, while also showing smaller variability in PR-AUC and ROC-AUC across positive-label budgets. Compared with DeepND, Deep-MOTIFs improved F1 by 10.8%, PR-AUC by 9.1%, and ROC-AUC by 9.5%. These gains indicate that the advantage of Deep-MOTIFs under reduced positive supervision is not simply due to using multi-omics features or network information, but reflects the benefit of empirical-prior calibration and PU ranking under incomplete ASD-positive annotations.

**Table 2:** Robustness to incomplete ASD-positive annotations. We evaluate model performance when progressively smaller fractions of known ASD-positive genes are available for training. Values summarize results across 40%, 60%, 80%, and 100% positive-supervision levels and are reported as mean*± *standard deviation. Higher values indicate better performance for all metrics

***ASD-specific gene-prioritization baselines***				
**Method**	**Accuracy**	**F1**	**PR-AUC**	**ROC-AUC**
**Deep-MOTIFs**	**0.7739*± *0.0055**	**0.7528*± *0.0073**	**0.8602*± *0.0036**	**0.8314*± *0.0038**
forecASD	0.7119*± *0.0050	0.7070*± *0.0110	0.7797*± *0.0146	0.7832*± *0.0082
FRN-RF	0.7356*± *0.0070	0.7276*± *0.0078	0.8253*± *0.0057	0.8032*± *0.0047
DeepND	0.6964*± *0.0205	0.6796*± *0.0080	0.7888*± *0.0171	0.7591*± *0.0114
DeepASDPred	0.5958*± *0.0235	0.5832*± *0.0763	0.6963*± *0.0040	0.6930*± *0.0057
Krishnan-SVM	0.6656*± *0.0060	0.6274*± *0.0266	0.7071*± *0.0068	0.7247*± *0.0066
***General-purpose machine-learning and deep-learning baselines***				
**Method**	**Accuracy**	**F1**	**PR-AUC**	**ROC-AUC**
DeepGBM	0.7385*± *0.0087	0.7298*± *0.0074	0.8393*± *0.0122	0.8121*± *0.0121
XGBoost	0.7424*± *0.0088	0.7307*± *0.0073	0.8343*± *0.0013	0.8098*± *0.0022
SAINT	0.7224*± *0.0197	0.7132*± *0.0164	0.8160*± *0.0218	0.7947*± *0.0183
FT-Transformer	0.7333*± *0.0198	0.6926*± *0.0424	0.8369*± *0.0060	0.8027*± *0.0055
TabNet	0.6769*± *0.0310	0.6740*± *0.0211	0.7404*± *0.0453	0.7287*± *0.0320

Bold values are highest value of all methods.

### Robustness to weak-reference label noise

In ASD risk-gene discovery, label uncertainty arises from both sides of the supervision process. Genes with strong ASD evidence provide reliable positive labels, but reference negatives are not definitive non-risk genes, and the known positive set remains incomplete as new genetic evidence continues to accumulate. We therefore evaluated robustness under two complementary label-contamination mechanisms. To model uncertainty in weak reference negatives, we randomly flipped 5%, 10%, or 20% of training negative labels to positive labels within each fold, representing the possibility that presumed non-risk genes may include undiscovered ASD risk genes. To model incomplete ASD-positive annotation, we randomly flipped 5%, 10%, or 20% of training positive labels to negative labels, thereby hiding known ASD genes from positive supervision. In both settings, perturbations were applied only to the training folds, while held-out folds were kept unchanged.

Figure 5 summarizes performance across the six label-contamination settings. Deep-MOTIFs achieved the highest average Accuracy, F1, PR-AUC, and ROC-AUC, with limited variation across perturbation settings. The separation from competing methods was most pronounced for PR-AUC and ROC-AUC, indicating that Deep-MOTIFs better preserves genome-wide discriminative ranking when training supervision is corrupted. Thus, the observed robustness is not limited to threshold-based classification, but extends to the ranking-oriented behavior required for candidate gene prioritization.

**Figure 5:**
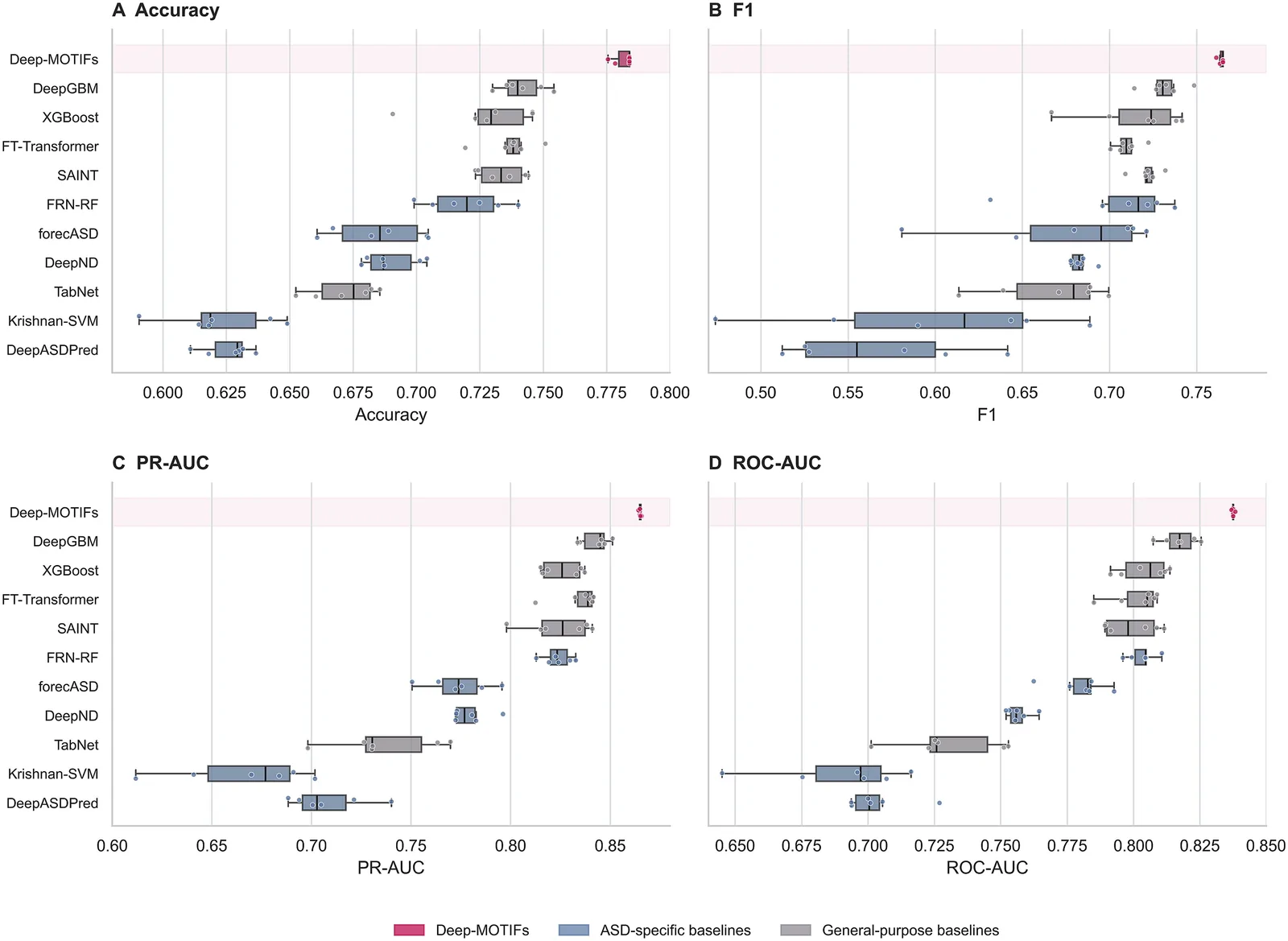
Overall robustness under label contamination of different methods. We summarize model performance across six label-noise settings: 5%, 10%, and 20% contamination of weak reference negatives, and 5%, 10%, and 20% contamination of ASD-positive labels. Bars denote mean performance across the six perturbation settings, and error bars denote standard deviation across settings. (A–D), Accuracy, F1, PR-AUC, and ROC-AUC. Deep-MOTIFs achieves the strongest average performance across all four metrics, indicating stable prediction and ranking quality under both weak-negative contamination and incomplete positive annotation.

Tables 3 and 4 provide the setting-specific results. Under weak-reference negative contamination, Deep-MOTIFs consistently outperformed forecASD across the 5%, 10%, and 20% perturbation levels, improving average F1, PR-AUC, ROC-AUC, and MCC-AUC by 6.7%, 13.4%, 8.3%, and 21.9%, respectively. Compared with DeepND, Deep-MOTIFs achieved gains of 11.3% in F1 and 29.4% in MCC-AUC. Under positive-label contamination, the advantage was even stronger: relative to forecASD, Deep-MOTIFs improved average F1, PR-AUC, ROC-AUC, and MCC-AUC by 20.4%, 10.2%, 6.5%, and 18.3%, and exceeded DeepND by 12.4%, 11.5%, 10.9%, and 30.1% on the same metrics. These results support the use of empirical-prior calibration and soft latent responsibilities, rather than hard positive–negative labels, for ASD risk-gene prioritization under weak and incomplete supervision.

**Table 3:** Robustness to contamination of weak reference negatives. In each training fold, we randomly selected 5%, 10%, or 20% of weak reference negative genes and flipped their labels from negative to positive, while leaving the held-out fold unchanged. This perturbation mimics the possibility that some reference negatives may in fact correspond to undiscovered ASD risk genes. Values denote mean performance across five-folds. Bold indicates the best result within each setting; higher values indicate better performance

***Clean***										
**Method**	**Classification**				**Top-ranked prioritization**			**MCC- and enrichment-based ranking**		
	**Accuracy**	**F1**	**PR-AUC**	**ROC-AUC**	**P@10**	**P@50**	**nDCG@50**	**MCC-AUC**	**Lift@50**	**MCC-max**
**Deep-MOTIFs**	**0.7812**	**0.7719**	**0.8649**	**0.8365**	**1.0000**	**0.9720**	0.9785	**0.3948**	**1.9441**	**0.5883**
forecASD	0.7109	0.7035	0.7961	0.7919	**1.0000**	0.9000	0.9217	0.3441	1.8000	0.4773
FRN-RF	0.7373	0.7261	0.8308	0.8079	**1.0000**	0.9520	0.9640	0.3654	1.9041	0.5093
DeepND	0.7143	0.6926	0.8145	0.7786	**1.0000**	0.9560	0.9672	0.3299	1.9121	0.4859
DeepASDPred	0.6468	0.6139	0.7128	0.7150	0.9000	0.8000	0.8207	0.2563	1.6001	0.3645
Krishnan-SVM	0.6704	0.6363	0.7155	0.7320	**1.0000**	0.9200	0.9342	0.2732	1.8400	0.4070
XGBoost	0.7317	0.7206	0.8359	0.8098	**1.0000**	0.9640	0.9741	0.3670	1.9280	0.5210
DeepGBM	0.7463	0.7385	0.8525	0.8249	**1.0000**	**0.9720**	**0.9797**	0.3834	**1.9441**	0.5408
TabNet	0.7013	0.6977	0.7723	0.7599	0.9000	0.8680	0.8769	0.3067	1.7361	0.4516
SAINT	0.7368	0.7226	0.8360	0.8126	0.9800	0.9640	0.9657	0.3687	1.9280	0.5272
FT-Transformer	0.7339	0.6991	0.8353	0.8006	**1.0000**	0.9640	0.9745	0.3553	1.9281	0.5150
***Negative contamination 5%***										
**Method**	**Classification**				**Top-ranked prioritization**			**MCC- and enrichment-based ranking**		
	**Accuracy**	**F1**	**PR-AUC**	**ROC-AUC**	**P@10**	**P@50**	**nDCG@50**	**MCC-AUC**	**Lift@50**	**MCC-max**
**Deep-MOTIFs**	**0.7840**	**0.7651**	**0.8648**	**0.8376**	**1.0000**	**0.9760**	**0.9811**	**0.3962**	**1.9521**	**0.5854**
forecASD	0.7041	0.7135	0.7723	0.7822	**1.0000**	0.8840	0.9063	0.3337	1.7680	0.4601
FRN-RF	0.7401	0.7375	0.8327	0.8107	**1.0000**	0.9480	0.9600	0.3688	1.8961	0.5206
DeepND	0.7041	0.6937	0.7962	0.7644	0.9800	0.9160	0.9274	0.3116	1.8321	0.4760
DeepASDPred	0.6300	0.5252	0.7006	0.7000	0.9000	0.7960	0.8164	0.2382	1.5921	0.3479
Krishnan-SVM	0.6490	0.6525	0.7018	0.7162	0.9800	0.9320	0.9444	0.2652	1.8640	0.4235
XGBoost	0.7458	0.7417	0.8372	0.8118	**1.0000**	0.9600	0.9706	0.3691	1.9201	0.5224
DeepGBM	0.7542	0.7486	0.8512	0.8255	**1.0000**	**0.9760**	0.9809	0.3830	**1.9521**	0.5526
TabNet	0.6800	0.6878	0.7698	0.7512	0.9600	0.8880	0.9120	0.3015	1.7760	0.4013
SAINT	0.7441	0.7206	0.8410	0.8115	**1.0000**	0.9680	0.9747	0.3668	1.9361	0.5279
FT-Transformer	0.7379	0.7005	0.8398	0.8056	**1.0000**	0.9640	0.9746	0.3611	1.9281	0.5247
***Negative contamination 10%***										
**Method**	**Classification**				**Top-ranked prioritization**			**MCC- and enrichment-based ranking**		
	**Accuracy**	**F1**	**PR-AUC**	**ROC-AUC**	**P@10**	**P@50**	**nDCG@50**	**MCC-AUC**	**Lift@50**	**MCC-max**
**Deep-MOTIFs**	**0.7756**	**0.7634**	**0.8642**	**0.8373**	**1.0000**	**0.9760**	**0.9816**	**0.3956**	**1.9521**	**0.5780**
forecASD	0.6822	0.7105	0.7638	0.7759	**1.0000**	0.8600	0.8902	0.3264	1.7200	0.4642
FRN-RF	0.7148	0.7272	0.8298	0.8046	**1.0000**	0.9440	0.9589	0.3626	1.8881	0.5064
DeepND	0.6783	0.6849	0.7807	0.7555	0.9800	0.9160	0.9217	0.3041	1.8321	0.4259
DeepASDPred	0.6181	0.6416	0.6883	0.6939	0.8600	0.7800	0.7898	0.2320	1.5602	0.3209
Krishnan-SVM	0.6192	0.6436	0.6909	0.6985	**1.0000**	0.9240	0.9427	0.2412	1.8480	0.3829
XGBoost	0.7312	0.7383	0.8352	0.8101	**1.0000**	0.9360	0.9532	0.3666	1.8720	0.5256
DeepGBM	0.7379	0.7269	0.8472	0.8177	**1.0000**	0.9680	0.9766	0.3766	1.9361	0.5263
TabNet	0.6822	0.6996	0.7634	0.7529	0.9600	0.8920	0.9066	0.3016	1.7840	0.4049
SAINT	0.7368	0.7208	0.8345	0.8045	**1.0000**	0.9640	0.9725	0.3594	1.9281	0.5217
FT-Transformer	0.7508	0.7224	0.8410	0.8089	**1.0000**	0.9680	0.9766	0.3649	1.9360	0.5330
***Negative contamination 20%***										
**Method**	**Classification**				**Top-ranked prioritization**			**MCC- and enrichment-based ranking**		
	**Accuracy**	**F1**	**PR-AUC**	**ROC-AUC**	**P@10**	**P@50**	**nDCG@50**	**MCC-AUC**	**Lift@50**	**MCC-max**
**Deep-MOTIFs**	**0.7784**	**0.7613**	**0.8649**	**0.8375**	**1.0000**	**0.9760**	0.9812	**0.3959**	**1.9521**	**0.5821**
forecASD	0.6608	0.7212	0.7506	0.7625	**1.0000**	0.8680	0.8995	0.3145	1.7359	0.4293
FRN-RF	0.6991	0.7219	0.8225	0.7960	**1.0000**	0.9560	0.9669	0.3522	1.9121	0.4874
DeepND	0.6867	0.6779	0.7731	0.7532	0.9200	0.9120	0.9170	0.3021	1.8241	0.4395
DeepASDPred	0.6109	0.6060	0.6938	0.6937	0.8800	0.8080	0.8108	0.2320	1.6161	0.3235
Krishnan-SVM	0.6142	0.6887	0.6838	0.7070	0.9800	0.8760	0.8922	0.2458	1.7522	0.4273
XGBoost	0.6906	0.7223	0.8185	0.7913	**1.0000**	0.9360	0.9521	0.3450	1.8720	0.4952
DeepGBM	0.7300	0.7143	0.8444	0.8170	**1.0000**	0.9720	0.9786	0.3753	1.9441	0.5219
TabNet	0.6524	0.6889	0.7303	0.7264	0.8600	0.8480	0.8459	0.2683	1.6961	0.3821
SAINT	0.7233	0.7091	0.8176	0.7914	0.9600	0.9440	0.9410	0.3413	1.8880	0.5023
FT-Transformer	0.7384	0.7116	0.8324	0.7955	**1.0000**	**0.9760**	**0.9826**	0.3495	**1.9521**	0.5072

Bold values are highest value of all methods.

**Table 4:** Robustness to positive-label contamination under incomplete ASD annotations. For each training fold, 5%, 10%, or 20% of ASD-positive labels were randomly contaminated by flipping them from positive to negative, while held-out folds were left unchanged. This perturbation mimics incomplete ASD-positive annotation, where true risk genes may be unavailable as positive supervision during training. Values denote mean performance across five-folds. Bold indicates the best result within each setting; higher values indicate better performance

***Clean***										
**Method**	**Classification**				**Top-ranked prioritization**			**MCC- and enrichment-based ranking**		
	**Accuracy**	**F1**	**PR-AUC**	**ROC-AUC**	**P@10**	**P@50**	**nDCG@50**	**MCC-AUC**	**Lift@50**	**MCC-max**
**Deep-MOTIFs**	**0.7812**	**0.7719**	**0.8649**	**0.8365**	**1.0000**	**0.9720**	0.9785	**0.3948**	**1.9441**	**0.5883**
forecASD	0.7109	0.7035	0.7961	0.7919	**1.0000**	0.9000	0.9217	0.3441	1.8000	0.4773
FRN-RF	0.7373	0.7261	0.8308	0.8079	**1.0000**	0.9520	0.9640	0.3654	1.9041	0.5093
DeepND	0.7143	0.6926	0.8145	0.7786	**1.0000**	0.9560	0.9672	0.3299	1.9121	0.4859
DeepASDPred	0.6468	0.6139	0.7128	0.7150	0.9000	0.8000	0.8207	0.2563	1.6001	0.3645
Krishnan-SVM	0.6704	0.6363	0.7155	0.7320	**1.0000**	0.9200	0.9342	0.2732	1.8400	0.4070
XGBoost	0.7317	0.7206	0.8359	0.8098	**1.0000**	0.9640	0.9741	0.3670	1.9280	0.5210
DeepGBM	0.7463	0.7385	0.8525	0.8249	**1.0000**	**0.9720**	**0.9797**	0.3834	**1.9441**	0.5408
TabNet	0.7013	0.6977	0.7723	0.7599	0.9000	0.8680	0.8769	0.3067	1.7361	0.4516
SAINT	0.7368	0.7226	0.8360	0.8126	0.9800	0.9640	0.9657	0.3687	1.9280	0.5272
FT-Transformer	0.7339	0.6991	0.8353	0.8006	**1.0000**	0.9640	0.9745	0.3553	1.9281	0.5150
***Positive hiding 5%***										
**Method**	**Classification**				**Top-ranked prioritization**			**MCC- and enrichment-based ranking**		
	**Accuracy**	**F1**	**PR-AUC**	**ROC-AUC**	**P@10**	**P@50**	**nDCG@50**	**MCC-AUC**	**Lift@50**	**MCC-max**
**Deep-MOTIFs**	**0.7840**	**0.7652**	**0.8654**	**0.8369**	**1.0000**	**0.9800**	**0.9841**	**0.3953**	**1.9601**	**0.5860**
forecASD	0.7047	0.6797	0.7957	0.7927	0.9600	0.9160	0.9292	0.3463	1.8320	0.4686
FRN-RF	0.7323	0.7110	0.8240	0.8047	**1.0000**	0.9520	0.9616	0.3620	1.9041	0.5011
DeepND	0.7013	0.6835	0.7724	0.7588	0.9600	0.8720	0.8955	0.3067	1.7441	0.4287
DeepASDPred	0.6367	0.5824	0.7402	0.7269	0.9200	0.8440	0.8737	0.2715	1.6880	0.3842
Krishnan-SVM	0.6423	0.5901	0.6698	0.6960	0.8600	0.9000	0.9024	0.2317	1.8000	0.3563
XGBoost	0.7458	0.7253	0.8330	0.8137	0.9600	0.9440	0.9540	0.3708	1.8880	0.5347
DeepGBM	0.7491	0.7369	0.8455	0.8229	**1.0000**	0.9640	0.9713	0.3806	1.9281	0.5403
TabNet	0.6856	0.6709	0.7264	0.7252	0.8600	0.8120	0.8186	0.2645	1.6241	0.4037
SAINT	0.7430	0.7321	0.8382	0.8087	**1.0000**	0.9680	0.9738	0.3642	1.9361	0.5280
FT-Transformer	0.7413	0.7128	0.8416	0.8079	**1.0000**	0.9680	0.9775	0.3632	1.9361	0.5329
***Positive hiding 10%***										
**Method**	**Classification**				**Top-ranked prioritization**			**MCC- and enrichment-based ranking**		
	**Accuracy**	**F1**	**PR-AUC**	**ROC-AUC**	**P@10**	**P@50**	**nDCG@50**	**MCC-AUC**	**Lift@50**	**MCC-max**
**Deep-MOTIFs**	**0.7840**	**0.7651**	**0.8657**	**0.8384**	**1.0000**	**0.9760**	**0.9813**	**0.3971**	**1.9521**	**0.5884**
forecASD	0.6890	0.6466	0.7856	0.7841	0.9600	0.9240	0.9283	0.3346	1.8481	0.4657
FRN-RF	0.7250	0.6960	0.8192	0.7993	0.9800	0.9360	0.9484	0.3555	1.8721	0.4888
DeepND	0.6873	0.6778	0.7827	0.7561	0.9800	0.9200	0.9376	0.3055	1.8401	0.4357
DeepASDPred	0.6316	0.5123	0.7213	0.7055	0.9200	0.8480	0.8676	0.2475	1.6961	0.3510
Krishnan-SVM	0.6181	0.5418	0.6409	0.6753	0.9000	0.7880	0.8260	0.2250	1.5760	0.3664
XGBoost	0.7278	0.6999	0.8151	0.8025	0.9800	0.9360	0.9377	0.3592	1.8720	0.4897
DeepGBM	0.7418	0.7287	0.8348	0.8125	0.9600	0.9600	0.9659	0.3683	1.9200	0.5206
TabNet	0.6704	0.6390	0.7307	0.7228	0.8200	0.8520	0.8505	0.2637	1.7043	0.3816
SAINT	0.7244	0.7249	0.8152	0.7894	0.9400	0.9440	0.9517	0.3412	1.8880	0.4946
FT-Transformer	0.7351	0.7076	0.8375	0.8045	**1.0000**	0.9680	0.9768	0.3592	1.9361	0.5286
***Positive hiding 20%***										
**Method**	**Classification**				**Top-ranked prioritization**			**MCC- and enrichment-based ranking**		
	**Accuracy**	**F1**	**PR-AUC**	**ROC-AUC**	**P@10**	**P@50**	**nDCG@50**	**MCC-AUC**	**Lift@50**	**MCC-max**
**Deep-MOTIFs**	**0.7840**	**0.7651**	**0.8652**	**0.8376**	**1.0000**	**0.9760**	**0.9811**	**0.3961**	**1.9521**	**0.5885**
forecASD	0.6670	0.5808	0.7754	0.7833	0.9400	0.8960	0.9026	0.3241	1.7920	0.4550
FRN-RF	0.7064	0.6317	0.8128	0.8044	0.9800	0.9080	0.9277	0.3609	1.8160	0.4954
DeepND	0.6805	0.6817	0.7728	0.7520	0.9400	0.9040	0.9187	0.3015	1.8081	0.4449
DeepASDPred	0.6288	0.5276	0.7049	0.7009	0.8600	0.8120	0.8200	0.2384	1.6241	0.3491
Krishnan-SVM	0.5906	0.4739	0.6118	0.6451	0.8800	0.8560	0.8673	0.1839	1.7118	0.2900
XGBoost	0.7233	0.6667	0.8161	0.7954	**1.0000**	0.9240	0.9387	0.3499	1.8480	0.5061
DeepGBM	0.7357	0.7324	0.8336	0.8074	**1.0000**	0.9560	0.9667	0.3630	1.9120	0.5106
TabNet	0.6603	0.6136	0.6982	0.7012	0.8200	0.7760	0.7833	0.2393	1.5519	0.3605
SAINT	0.7300	0.7220	0.7980	0.7892	0.8800	0.9000	0.8980	0.3375	1.8000	0.4966
FT-Transformer	0.7193	0.7062	0.8124	0.7851	0.9800	0.9520	0.9596	0.3371	1.9041	0.4924

Bold values are highest value of all methods.

### Sensitivity analysis of hyper-parameters

Figure 6 evaluates the robustness of Deep-MOTIFs to hyper-parameters that control the latent-responsibility weighting, PU ranking objective, and final prior–posterior rank fusion. We considered the entropy-shrinkage coefficient $\delta \in \{0.20,0.40,0.50,0.55,0.60,0.80,1.00\}$, PU class-prior weight $\pi \in \{0.01,0.03,0.05,0.06,0.10\}$, minimum negative-responsibility floor $w_{\min }\in \{0.05,0.10,0.20,0.30\}$, pairwise ranking weight $\lambda _{\mathrm{rank}}\in \{0.25,0.50,0.75,1.00\}$, prior-fusion weight $\lambda _{\mathrm{prior}}\in \{0.25,0.50,0.75,1.00\}$, and reciprocal-rank fusion constant $k_{\mathrm{rrf}}\in \{20,40,60,80,100\}$, with all other settings fixed at their default values. Overall performance remained stable across these controlled ranges. PR-AUC varied from 0.8600 to 0.8646 for *δ *, 0.8648 to 0.8689 for *π *, 0.8654 to 0.8689 for $w_{\min }$, 0.8658 to 0.8688 for $\lambda _{\mathrm{rank}}$, 0.8623 to 0.8687 for $\lambda _{\mathrm{prior}}$, and 0.8685 to 0.8693 for $k_{\mathrm{rrf}}$. ROC-AUC showed a similarly narrow range, spanning 0.8302–0.8350 for *δ * and 0.8335–0.8409 across the remaining hyper-parameters. The selected configuration, *δ =0.60*, *π =0.10*, $w_{\min }=0.10$, $\lambda _{\mathrm{rank}}=0.50$, $\lambda _{\mathrm{prior}}=0.50$, and $k_{\mathrm{rrf}}=60$, therefore lies in a broad high-performing region.

**Figure 6:**
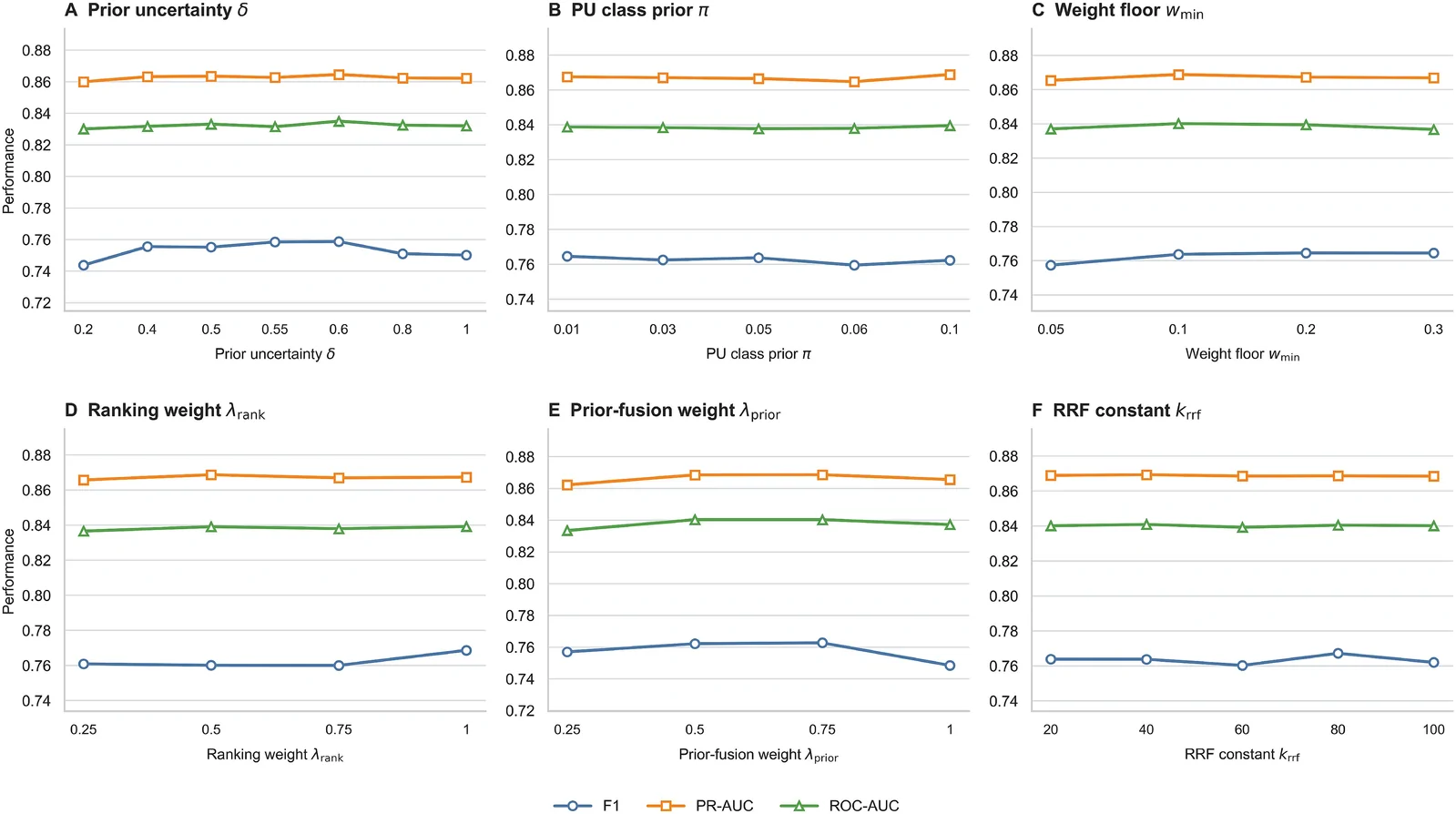
Hyper-parameter sensitivity analysis of Deep-MOTIFs. Performance of Deep-MOTIFs across six main hyper-parameters governing entropy-shrunk negative responsibility, PU class-prior weighting, minimum responsibility flooring, pairwise ranking, prior–posterior fusion, and reciprocal-rank fusion. Performance is summarized using F1, PR-AUC, and ROC-AUC.

### Ablation analysis of input data and methodological components

To better understand the independent contribution of each methodological component and input evidence view, we performed an ablation analysis covering 12 variants. Specifically, we evaluated variants that removed the empirical prior, disabled entropy shrinkage, removed prior-guided PU weighting, removed the pairwise PU ranking loss, used the posterior score alone, used the empirical prior score alone, replaced reciprocal-rank fusion with score-level fusion strategies, or restricted the model to genetic, BrainSpan, STRING, or BrainSpan+STRING evidence .

As shown in Table 5, Full Deep-MOTIFs achieved the highest F1, PR-AUC, and ROC-AUC among the ablation variants. Removing the empirical prior produced the largest degradation, reducing F1 by 0.0379, PR-AUC by 0.0287, and ROC-AUC by 0.0324, indicating that empirical-prior calibration provides essential guidance when reliable negative labels are unavailable. Using either the posterior score alone or the prior score alone also reduced performance, supporting the complementarity between the learned PU posterior and the empirical prior. Replacing reciprocal-rank fusion with score-level average or tuned-alpha fusion further decreased all three metrics, suggesting that rank-space fusion provides a more stable way to combine heterogeneous prior and posterior evidence.

**Table 5:** Component ablation analysis of Deep-MOTIFs. Each variant is evaluated over three random seeds with five-fold cross-validation. Values are reported as mean *± * standard deviation, with absolute changes relative to Full Deep-MOTIFs shown in parentheses

**Variant**	**F1**	**PR-AUC**	**ROC-AUC**
**Full Deep-MOTIFs**	**0.7564 *± * 0.0037**	**0.8624 *± * 0.0017**	**0.8339 *± * 0.0030**
w/o empirical prior	0.7185 *± * 0.0111 ($\color{red}{\downarrow 0.0379}$)	0.8337 *± * 0.0043 ($\color{red}{\downarrow 0.0287}$)	0.8015 *± * 0.0030 ($\color{red}{\downarrow 0.0324}$)
w/o entropy shrinkage	0.7424 *± * 0.0130 ($\color{red}{\downarrow 0.0140}$)	0.8582 *± * 0.0026 ($\color{red}{\downarrow 0.0042}$)	0.8256 *± * 0.0031 ($\color{red}{\downarrow 0.0083}$)
w/o prior-guided PU weighting	0.7508 *± * 0.0101 ($\color{red}{\downarrow 0.0056}$)	0.8579 *± * 0.0027 ($\color{red}{\downarrow 0.0045}$)	0.8273 *± * 0.0029 ($\color{red}{\downarrow 0.0066}$)
w/o pairwise PU ranking loss	0.7467 *± * 0.0074 ($\color{red}{\downarrow 0.0097}$)	0.8585 *± * 0.0054 ($\color{red}{\downarrow 0.0039}$)	0.8264 *± * 0.0071 ($\color{red}{\downarrow 0.0075}$)
posterior score alone	0.7275 *± * 0.0079 ($\color{red}{\downarrow 0.0289}$)	0.8420 *± * 0.0009 ($\color{red}{\downarrow 0.0204}$)	0.8103 *± * 0.0022 ($\color{red}{\downarrow 0.0236}$)
prior score alone	0.7237 *± * 0.0078 ($\color{red}{\downarrow 0.0327}$)	0.8401 *± * 0.0088 ($\color{red}{\downarrow 0.0223}$)	0.8084 *± * 0.0096 ($\color{red}{\downarrow 0.0255}$)
score-level average fusion	0.7278 *± * 0.0141 ($\color{red}{\downarrow 0.0286}$)	0.8529 *± * 0.0064 ($\color{red}{\downarrow 0.0095}$)	0.8232 *± * 0.0067 ($\color{red}{\downarrow 0.0107}$)
score-level tuned-alpha fusion	0.7253 *± * 0.0063 ($\color{red}{\downarrow 0.0311}$)	0.8483 *± * 0.0023 ($\color{red}{\downarrow 0.0141}$)	0.8181 *± * 0.0031 ($\color{red}{\downarrow 0.0158}$)
Genetic only	0.7299 *± * 0.0031 ($\color{red}{\downarrow 0.0265}$)	0.8420 *± * 0.0039 ($\color{red}{\downarrow 0.0204}$)	0.8061 *± * 0.0027 ($\color{red}{\downarrow 0.0278}$)
BrainSpan only	0.6934 *± * 0.0024 ($\color{red}{\downarrow 0.0630}$)	0.7263 *± * 0.0034 ($\color{red}{\downarrow 0.1361}$)	0.7334 *± * 0.0017 ($\color{red}{\downarrow 0.1005}$)
STRING only	0.6739 *± * 0.0055 ($\color{red}{\downarrow 0.0825}$)	0.5789 *± * 0.0096 ($\color{red}{\downarrow 0.2835}$)	0.6034 *± * 0.0054 ($\color{red}{\downarrow 0.2305}$)
BrainSpan + STRING	0.7112 *± * 0.0039 ($\color{red}{\downarrow 0.0452}$)	0.7711 *± * 0.0076 ($\color{red}{\downarrow 0.0913}$)	0.7648 *± * 0.0040 ($\color{red}{\downarrow 0.0691}$)

Downward arrows indicate the absolute decrease from Full Deep-MOTIFs for the corresponding metric.

Bold values are highest value of all methods.

The effects of entropy shrinkage, prior-guided PU weighting, and pairwise PU ranking were smaller in magnitude, but they address how strongly uncertain unlabeled genes are penalized as negatives during PU training. As an empirical diagnostic, we evaluated the fold-local prior and induced negative-responsibility weights on held-out reference labels. Held-out ASD-positive genes received substantially lower negative-responsibility weights than weak reference negatives (0.2260 versus 0.4911 on average), and 77.3% of held-out positives had $\widetilde{w}^{-}\leq 0.3$. This pattern is consistent with the intended role of entropy shrinkage, reducing false-negative pressure on ASD-like or prior-ambiguous genes while retaining negative evidence from weak reference negatives.

The view-level ablations further show that genetic association evidence is the strongest individual input source, with the genetic-only model achieving F1, PR-AUC, and ROC-AUC values of 0.7299, 0.8420, and 0.8061, respectively. Nevertheless, the full multi-view model outperformed the genetic-only variant across all three metrics, indicating that BrainSpan developmental expression and STRING network context add complementary information beyond genetic evidence alone. BrainSpan alone and STRING alone were weaker than the others; combining BrainSpan and STRING improved over either non-genetic view alone, supporting their complementary role in the multi-view architecture.

## Deep-MOTIFs prioritizes autism risk genes genome-wide

### Novel Deep-MOTIFs candidates converge on autism spectrum disorder-relevant biological mechanisms

To determine whether the highest-priority novel candidates nominated by Deep-MOTIFs carry biological signals beyond those provided by the curated training labels, we performed functional enrichment analysis on the top 200 novel genes. Novel candidates were defined as the highest-ranked genes after removing SFARI Category 1 and 2 genes used as positive labels during training. Enrichment was assessed using g:Profiler [59] with the full set of 14 331 genome-wide ranked genes as the background universe. At a Bonferroni-corrected significance threshold of 0.05, the top novel candidates yielded 660 significantly enriched terms across GO, HPO, KEGG, and Reactome, including 109 terms directly related to ASD or neurodevelopmental biology. Representative terms are summarized in Table 6 and reveal convergence on four major biological axes: ASD-related clinical phenotypes, synaptic function and neurotransmission, neuronal development, and chromatin-mediated gene regulation.

**Table 6:** Functional enrichment of top-ranked novel Deep-MOTIFs candidates. Enrichment analysis was performed for the top 200 novel Deep-MOTIFs candidates using g:Profiler, with all 14 331 genome-wide ranked genes used as the statistical background. Novel candidates were defined as the highest-ranked genes after excluding SFARI Categories 1 and 2 genes used as positive training labels. Representative Bonferroni-significant terms are grouped by biological theme. Overlap denotes the number of annotated query genes assigned to each term, with the corresponding background term size shown in parentheses

**Enriched term**	**Source**	**Biological theme**	**Overlap**	**Adjusted** *P*
*ASD and neurodevelopmental phenotype*				
Autistic behavior	HP	ASD/neurodevelopmental phenotype	48/198 (345/14 331)	$2.08\times 10^{-31}$
Intellectual disability	HP	ASD/neurodevelopmental phenotype	77/198 (1329/14 331)	$2.84\times 10^{-26}$
Neurodevelopmental delay	HP	ASD/neurodevelopmental phenotype	85/198 (1733/14 331)	$1.46\times 10^{-24}$
Restricted or repetitive behaviors or interests	HP	ASD/neurodevelopmental phenotype	25/198 (158/14 331)	$2.29\times 10^{-16}$
*Synaptic function and neurotransmission*				
Synaptic signaling	GO:BP	Synaptic function	65/198 (550/14 331)	$4.96\times 10^{-8}$
Synapse	GO:CC	Synaptic function	91/198 (1173/14 331)	$1.13\times 10^{-9}$
Transmission across chemical synapses	REAC	Synaptic function	14/198 (171/14 331)	$4.76\times 10^{-5}$
GABAergic synapse	KEGG	Synaptic function	8/198 (59/14 331)	$1.39\times 10^{-4}$
*Neuronal development and brain morphology*				
Neuron projection	GO:CC	Neuronal development	76/198 (904/14 331)	$3.53\times 10^{-10}$
Abnormal cerebral morphology	HP	Neuronal development	77/198 (1486/14 331)	$4.54\times 10^{-23}$
Nervous system development	GO:BP	Neuronal development	120/198 (1837/14 331)	$3.57\times 10^{-5}$
Axon guidance	KEGG	Neuronal development	11/198 (144/14 331)	$5.05\times 10^{-4}$
*Chromatin and transcriptional regulation*				
Chromatin organization	REAC	Chromatin regulation	11/198 (133/14 331)	$1.02\times 10^{-3}$
ATP-dependent chromatin remodeling	KEGG	Chromatin regulation	6/198 (68/14 331)	$3.40\times 10^{-2}$

The strongest enrichments correspond to phenotypic and neurodevelopmental annotations highly relevant to ASD. The HPO term *Autistic behavior* was the most significant ASD-specific annotation among the novel candidates ($P_{\mathrm{adj}} = 2.08\times 10^{-31}$), with 48 of 198 annotated query genes assigned to this term. Additional highly significant HPO enrichments included *Intellectual disability* ($P_{\mathrm{adj}} = 2.84\times 10^{-26}$), *Neurodevelopmental delay* ($P_{\mathrm{adj}} = 1.46\times 10^{-24}$), and *Restricted or repetitive behaviors or interests* ($P_{\mathrm{adj}} = 2.29\times 10^{-16}$). These terms span core clinical and developmental features frequently observed in ASD, suggesting that the top-ranked novel candidates are not generic high-scoring genes but are concentrated in loci with phenotypic relevance to neurodevelopmental disorders.

At the molecular and cellular levels, the novel candidates show strong enrichment for synaptic and neuronal mechanisms. Terms related to synaptic architecture and signaling were consistently overrepresented, including *Synapse* (GO:CC; $P_{\mathrm{adj}} = 1.13\times 10^{-9}$), *Synaptic signaling* (GO:BP; $P_{\mathrm{adj}} = 4.96\times 10^{-8}$), and *Transmission across chemical synapses* (Reactome; $P_{\mathrm{adj}} = 4.76\times 10^{-5}$). Enrichment for *GABAergic synapse* (KEGG; $P_{\mathrm{adj}} = 1.39\times 10^{-4}$) further indicates that inhibitory neurotransmission contributes to the biological signal captured by Deep-MOTIFs. In parallel, terms reflecting neuronal structure and development, including *Neuron projection* (GO:CC; $P_{\mathrm{adj}} = 3.53\times 10^{-10}$), *Abnormal cerebral morphology* (HPO; $P_{\mathrm{adj}} = 4.54\times 10^{-23}$), *Nervous system development* (GO:BP; $P_{\mathrm{adj}} = 3.57\times 10^{-5}$), and *Axon guidance* (KEGG; $P_{\mathrm{adj}} = 5.05\times 10^{-4}$), support a broader neurodevelopmental interpretation of the prioritized candidate set.

Finally, Deep-MOTIFs-prioritized novel genes are enriched for chromatin and transcriptional regulatory mechanisms, including *Chromatin organization* (Reactome; $P_{\mathrm{adj}} = 1.02\times 10^{-3}$) and *ATP-dependent chromatin remodeling* (KEGG; $P_{\mathrm{adj}} = 3.40\times 10^{-2}$). This signal is consistent with convergent evidence implicating chromatin remodeling and transcriptional regulation in ASD etiology [30]. Taken together, these enrichment patterns provide training-label-independent biological support for the Deep-MOTIFs ranking: after removing known SFARI-positive genes used for supervision, the highest-ranked novel candidates remain strongly concentrated in ASD-relevant phenotypic, synaptic, neurodevelopmental, and regulatory pathways. This mechanistic convergence supports the utility of Deep-MOTIFs as a genome-wide prioritization framework for nominating biologically plausible ASD candidate genes for downstream validation.

### The Deep-MOTIFs ranking captures a genome-wide autism spectrum disorder-relevant biological gradient

To evaluate whether the Deep-MOTIFs genome-wide ranking captures biological structure beyond the recovery of labeled ASD genes, we divided all 14 331 ranked genes into 10 equal-sized deciles and quantified the overlap of each decile with six representative ASD-related biological terms (Fig. 7). These terms were selected to cover complementary levels of ASD biology, including clinical phenotype annotation, synaptic function, neuronal architecture, chromatin regulation, and inhibitory neurotransmission. This analysis provides an orthogonal validation of the ranking: rather than asking whether Deep-MOTIFs predicts held-out labeled genes, it examines whether the full genome-wide ordering is enriched, in a graded manner, for biological processes and cellular components known to be relevant to ASD. For each decile, we computed the percentage of genes annotated to each term using curated gene sets from HPO, GO (biological process and cellular component), Reactome, and KEGG; the six terms were selected to represent distinct and complementary axes of ASD-relevant biology with the strongest and most interpretable decile-wise gradients.

**Figure 7:**
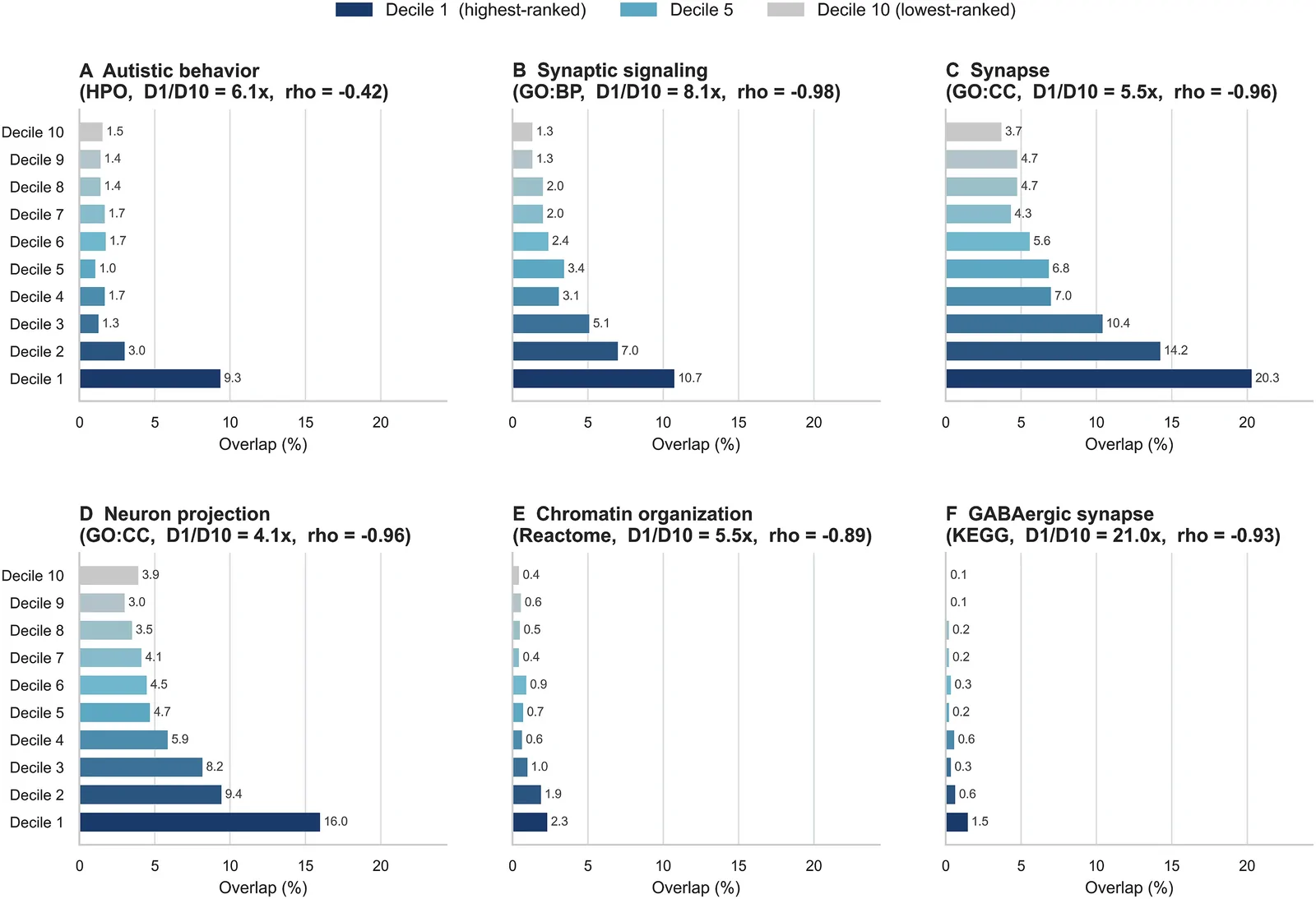
Genome-wide ASD-relevant biological gradient captured by Deep-MOTIFs. All ranked genes were divided into 10 equal-sized deciles according to the five-run mean Deep-MOTIFs score, with Decile 1 containing the highest-priority genes. (A–F), Decile-wise overlap with representative ASD-related biological terms spanning clinical phenotype annotation, synaptic function, neuronal architecture, chromatin regulation, and GABAergic neurotransmission. Each panel reports the Decile-1-to-Decile-10 enrichment ratio and the Spearman correlation between decile index and overlap percentage. A consistent decline from top- to bottom-ranked deciles indicates that Deep-MOTIFs organizes genome-wide candidates along an ASD-relevant biological gradient.

Deep-MOTIFs exhibits a coherent biological gradient across the genome-wide ranking. The strongest and most monotone patterns are observed for mechanistic annotations closely related to ASD neurobiology. For *Synaptic signaling*, 10.7% of genes in the highest-ranked decile are annotated to this GO biological-process term, compared with 1.3% in the lowest-ranked decile, corresponding to an 8.1-fold Decile-1-to-Decile-10 enrichment ratio and a strong negative decile-wise correlation (Spearman *ρ =-0.98*). Similar gradients are observed for *Synapse* (GO:CC; 5.5-fold, *ρ =-0.96*) and *Neuron projection* (GO:CC; 4.1-fold, *ρ =-0.96*), indicating that genes involved in synaptic and neuronal structures are systematically concentrated near the top of the Deep-MOTIFs ranking. The *GABAergic synapse* term shows the sharpest top-versus-bottom contrast, with overlap decreasing from 1.5% in Decile 1 to 0.07% in Decile 10, yielding a 21.0-fold enrichment ratio and a strong monotone trend (*ρ =-0.93*). This pattern is consistent with extensive evidence implicating GABAergic inhibitory dysfunction in ASD pathophysiology [60, 61]. In addition, *Chromatin organization* shows a 5.5-fold gradient (*ρ =-0.89*), supporting the ability of Deep-MOTIFs to prioritize genes involved in chromatin-regulatory mechanisms that have been repeatedly implicated in ASD risk [30].

The phenotype-level HPO term *Autistic behavior* is also enriched among top-ranked genes, with 9.3% overlap in Decile 1 compared with 1.5% in Decile 10, corresponding to a 6.1-fold enrichment ratio. Its weaker monotonicity across deciles (*ρ =-0.42*) is biologically and annotation-wise expected, because phenotype annotations are typically assigned to genes with established clinical evidence and therefore tend to be concentrated among the highest-confidence candidates rather than distributed smoothly across the genome. In contrast, the stronger monotone gradients observed for synaptic, neuronal, chromatin-regulatory, and GABAergic terms suggest that Deep-MOTIFs is not merely reproducing existing clinical annotations, but is organizing candidate genes according to broader mechanistic axes relevant to ASD biology.

## Conclusion

This study highlights incomplete supervision as a central obstacle in genome-wide ASD risk-gene prioritization. Curated ASD genes provide high-confidence positive labels, but reference negatives are uncertain and unlabeled genome-wide candidates may include undiscovered ASD risk genes. Deep-MOTIFs addresses this setting by combining empirical ASD-risk priors, latent-responsibility PU learning, and multi-view neural integration of genetic association, developmental brain transcriptomic, and protein-interaction evidence. The resulting rankings consistently improved over ASD-specific, PU, and general-purpose baselines, remained stable under reduced positive-label supervision and label contamination, and preserved strong performance among top-ranked candidates. These results indicate that modeling label uncertainty is not only statistically appropriate for ASD gene discovery, but also practically beneficial for genome-wide prioritization. The biological analyses further support this interpretation: top-ranked candidates were enriched for ASD-related phenotypic annotations and showed graded genome-wide concentration of synaptic, neurodevelopmental, GABAergic, and chromatin-regulatory signals.

Importantly, Deep-MOTIFs is not restricted to autism research; it is broadly applicable to the study of other complex traits and diseases. The primary requirement for this framework is an input dataset comprising both high-confidence positive labels (e.g. curated SFARI risk genes) and weak reference negatives (e.g. unconfirmed non-risk genes from independent studies). Additionally, users must gather relevant omics data for the target trait/disease, though incorporating all three omics layers, as was done in this study, is not strictly required.

Several limitations and future directions remain. First, the curated ASD-positive set is constrained by current SFARI annotation and will continue to evolve as sequencing cohorts expand, making prospective validation against newly reported ASD genes an important next step. Second, Deep-MOTIFs integrates genetic association, developmental transcriptomic, and protein-interaction evidence at the gene level, but does not directly model variant-level effects, isoform-specific regulation, or cell-state-specific mechanisms. Future extensions incorporating epigenomic, regulatory, single-cell, and cell-type-specific resources may provide additional resolution for interpreting these mechanisms. Third, systematic view-level interpretation could clarify how genetic, transcriptomic, and network evidence contribute to individual high-ranking candidates. Together with experimental follow-up of prioritized genes, these extensions may further strengthen the biological interpretation and practical utility of Deep-MOTIFs.

**Key Points**

- We proposed a deep learning framework, called Deep-MOTIFs, of integrating variant-level, gene level and protein level information to infer autism risk genes.
- We formulate autism spectrum disorder risk-gene prioritization as a positive-unlabeled (PU) ranking problem under incomplete supervision from curated positives, weak reference negatives, and genome-wide unlabeled candidates.
- We propose empirical-prior-guided entropy-regularized negative-responsibility weights and a risk-ordering formulation for responsibility-weighted PU learning.
- We compared Deep-MOTIFs to 13 other methods to demonstrate the better performance.

## Figures and Tables

**Figure A1:**
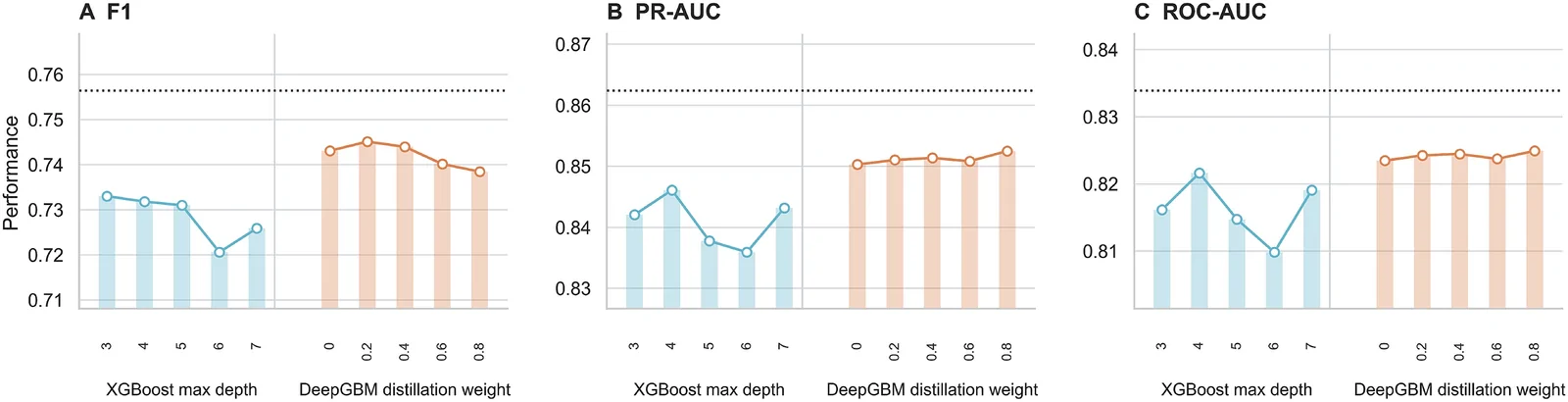
Robust comparison with tuned tree-based baselines. We varied the maximum tree depth for XGBoost and the GBDT distillation weight for DeepGBM while keeping all other hyper-parameters fixed. The dotted horizontal line denotes the performance of Deep-MOTIFs under the same held-out evaluation protocol.

## Data Availability

All datasets are publicly available. TADA information is from https://www.nature.com/articles/s41588-022-01104-0; WGS FDR https://www.sciencedirect.com/science/article/pii/S0092867422013241; BrainSpan https://www.nature.com/articles/nature10523; STRING https://string-db.org/,andSFARIhttps://www.sfari.org/.

## Appendix

### Use of AI and AI-assisted Technologies

During the preparation of this work, the authors used AI-assisted tools to generate part of illustrative figures and polish the writings. The authors reviewed and edited all these contents as needed and take full responsibility for the content of the publication.

### Statistical and algorithmic details of Deep-MOTIFs

#### Positive-unlabeled formulation and entropy-calibrated responsibility weighting

##### Definition

We formulate Deep-MOTIFs as a PU ranking problem under weak reference supervision. Let $\mathcal{G}=\{1,\ldots ,N\}$ denotes the set of aligned genome-wide candidate genes. For each gene $g\in \mathcal{G}$, let $X_{g}$ denotes its observed multi-omics feature representation and let $Y_{g}\in \{0,1\}$ denotes its unobserved ASD-risk status, where $Y_{g}=1$ indicates an ASD risk gene. The observed supervision is represented by an evidence state $S_{g}\in \{P_{L},N_{R},U\}$, where $P_{L}$ denotes curated ASD-positive genes, $N_{R}$ denotes weak reference negatives, and *U* denotes genome-wide unlabeled candidate genes. We write $n_{P}=|P_{L}|$, $n_{R}=|N_{R}|$, and $n_{U}=|U|$. Genes in *U* are not treated as negatives; instead, they form an unlabeled mixture that may contain undiscovered ASD risk genes:

(A.1)
\begin{align*}& \begin{aligned} p(X_{g}\mid S_{g}=U) &= \alpha\,p(X_{g}\mid Y_{g}=1,S_{g}=U)\\ & + (1-\alpha)\,p(X_{g}\mid Y_{g}=0,S_{g}=U), \end{aligned}\end{align*}

where $\alpha =P(Y_{g}=1\mid S_{g}=U)$ is unknown.

Deep-MOTIFs learns gene-level ASD evidence through an empirical-prior stage and a PU posterior-ranking stage. As shown in Equation (1), the complement $1-\hat{\rho }(g)$ provides an empirical estimate of the latent negative responsibility. For an unlabeled gene, the ideal negative responsibility is

(A.2)
\begin{align*}& w_{g}^{-*} = P(Y_{g}=0\mid X_{g},S_{g}=U),\end{align*}

which is unobserved and therefore cannot be used directly in training.

Let $f(X_{g})$ denotes a generic model score used in the PU risk analysis, where larger values indicate stronger ASD-relevant evidence. In the Deep-MOTIFs architecture, the PU posterior score is denoted by $s_{\mathrm{PU}}(g)$, and the final fused genome-wide score after prior–posterior rank-space fusion is denoted by $S_{\mathrm{final}}(g)$. Let $\ell (f(X_{g}),y)$ be a non-negative loss for target $y\in \{0,1\}$, and let $\pi \in (0,1)$ denotes the PU class-prior weight for curated positives. The responsibility-weighted PU risk can be written as

(A.3)
\begin{align*}& R_{\mathrm{PU}}^{*}(f) = \pi\,\mathbb{E}_{g\in P_{L}} \left\{ \ell(f(X_{g}),1) \right\} + \mathbb{E}_{g\in U} \left\{ w_{g}^{-*}\ell(f(X_{g}),0) \right\}.\end{align*}

Because $w_{g}^{-*}$ is unobserved, a direct empirical-Bayes approximation replaces it by $1-\hat{\rho }(g)$:

(A.4)
\begin{align*}& R_{\mathrm{EB}}(f) = \pi\,\mathbb{E}_{g\in P_{L}} \left\{ \ell(f(X_{g}),1) \right\} + \mathbb{E}_{g\in U} \left\{ \bigl(1-\hat{\rho}(g)\bigr)\ell(f(X_{g}),0) \right\}.\end{align*}

However, this point estimate can over-penalize unlabeled genes with uncertain prior evidence. We therefore shrink the empirical negative-responsibility score according to prior uncertainty.

Define the binary entropy of the calibrated empirical prior as

(A.5)
\begin{align*}& H(\hat{\rho}(g)) = -\hat{\rho}(g)\log \hat{\rho}(g) - \bigl(1-\hat{\rho}(g)\bigr) \log\bigl(1-\hat{\rho}(g)\bigr).\end{align*}

For a shrinkage parameter $\delta \geq 0$, the entropy-induced uncertainty radius is $\delta H(\hat{\rho }(g))$. This defines the following entropy-calibrated responsibility interval:

(A.6)
\begin{align*}& \begin{aligned} w_{g,L}^{-} &= \max\left\{ 0,\, 1-\hat{\rho}(g) - \delta H(\hat{\rho}(g)) \right\}, \\ w_{g,U}^{-} &= \min\left\{ 1,\, 1-\hat{\rho}(g) + \delta H(\hat{\rho}(g)) \right\}. \end{aligned}\end{align*}

The interval is wider for genes with ambiguous prior evidence and narrower for genes whose empirical prior is close to 0 or 1.

##### Risk ordering under responsibility uncertainty

For any negative-responsibility assignment $w^{-}(g)$ satisfying $w^{-}(g)\in [w_{g,L}^{-},w_{g,U}^{-}]$ for all *g∈ U*, define the corresponding responsibility-weighted PU risk as

(A.7)
\begin{align*}& R(f;w^{-}) = \pi\,\mathbb{E}_{g\in P_{L}} \left\{ \ell(f(X_{g}),1) \right\} + \mathbb{E}_{g\in U} \left\{ w^{-}(g)\ell(f(X_{g}),0) \right\}.\end{align*}

If $\ell (f(X_{g}),0)\geq 0$, then

(A.8)
\begin{align*}& R_{L}(f) \leq R(f;w^{-}) \leq R_{U}(f),\end{align*}

where

(A.9)
\begin{align*}& R_{L}(f) = \pi\,\mathbb{E}_{g\in P_{L}} \left\{ \ell(f(X_{g}),1) \right\} + \mathbb{E}_{g\in U} \left\{ w_{g,L}^{-}\ell(f(X_{g}),0) \right\},\end{align*}

and

(A.10)
\begin{align*}& R_{U}(f) = \pi\,\mathbb{E}_{g\in P_{L}} \left\{ \ell(f(X_{g}),1) \right\} + \mathbb{E}_{g\in U} \left\{ w_{g,U}^{-}\ell(f(X_{g}),0) \right\}.\end{align*}

This ordering follows directly from $w_{g,L}^{-}\leq w^{-}(g)\leq w_{g,U}^{-}$ and the non-negativity of $\ell (f(X_{g}),0)$, which preserve the ordering after multiplication by the unlabeled negative-loss term and expectation over *U*.

In implementation, Deep-MOTIFs uses the lower-side entropy-shrunk responsibility as a conservative training weight, with a small floor to avoid degeneracy:

(A.11)
\begin{align*}& \widetilde{w}^{-}(g) = \mathrm{clip}_{[w_{\min},1]} \left( 1-\hat{\rho}(g) - \delta H(\hat{\rho}(g)) \right).\end{align*}

Here, $w_{\min }\geq 0$ prevents the unlabeled negative contribution from vanishing. Genes with confidently low ASD prior receive larger negative weights, whereas genes with ambiguous or ASD-like prior evidence contribute more conservatively to PU learning.

#### Neural network architecture for Deep-MOTIFs

The Stage-II module in Deep-MOTIFs is a multi-view PU posterior ranker that refines the empirical prior by learning complementary evidence from genetic, developmental, and network-derived representations. For each gene *g*, the multi-omics input is decomposed as $ X_{g}= \left ( X_{g}^{\mathrm{gene}}, X_{g}^{\mathrm{brain}}, X_{g}^{\mathrm{string}} \right )$, where $X_{g}^{\mathrm{gene}}$ denotes gene-level tabular evidence, $X_{g}^{\mathrm{brain}}\in \mathbb{R}^{16\times 50}$ denotes the BrainSpan developmental expression matrix, and $X_{g}^{\mathrm{string}}$ denotes STRING-derived network features. Each view is mapped into tokens with a common embedding dimension *d*.

The gene-level view is encoded by a full-vector MLP tokenizer. Specifically, $X_{g}^{\mathrm{gene}}$ is passed through two linear layers with hidden dimension *\max (2d,64)*, layer normalization, GELU nonlinearities, and dropout, yielding a single gene token

(A.12)
\begin{align*}& z_{g}^{\mathrm{gene}} = \mathrm{MLP}_{\mathrm{gene}} \left( X_{g}^{\mathrm{gene}} \right) \in \mathbb{R}^{1\times d}.\end{align*}

The BrainSpan encoder preserves the structured *16× 50* developmental organization. For each brain region, a temporal convolutional branch applies two 1D convolutional layers with GELU activations, followed by adaptive average pooling and layer normalization, producing 16 region-level developmental tokens. In parallel, a two-layer bidirectional LSTM processes the time-major BrainSpan representation, where each developmental time point contains expression across all 16 regions. The BiLSTM hidden size is *\max (d/2,16)*, and its bidirectional output is projected to dimension *d* through a linear layer, layer normalization, GELU activation, and dropout. The resulting global developmental token is gated into the region-level tokens:

(A.13)
\begin{align*}& z_{g}^{\mathrm{brain}} = \left[ z_{g}^{\mathrm{global}}; z_{g,1}^{\mathrm{region}}, \ldots, z_{g,16}^{\mathrm{region}} \right] \in \mathbb{R}^{17\times d}.\end{align*}

STRING evidence is first smoothed over the weighted protein-interaction graph before neural tokenization. Let $X_{g}^{\mathrm{string},(0)}=X_{g}^{\mathrm{string}}$ denote the initial STRING-derived feature vector. For each fold, graph smoothing is restricted to the fold-specific training universe $\mathcal{A}$, so that no held-out label information is used during feature propagation. Given normalized STRING edge weights $w_{gj}\in (0,1]$, Deep-MOTIFs applies $L_{\mathrm{str}}$ layers of local weighted neighbor aggregation:

(A.14)
\begin{align*}& \begin{aligned} X_{g}^{\mathrm{string},(l+1)} &= \lambda X_{g}^{\mathrm{string},(l)} + (1-\lambda) \frac{ \sum_{j\in \mathcal{N}(g)\cap \mathcal{A}} w_{gj}X_{j}^{\mathrm{string},(l)} }{ \sum_{j\in \mathcal{N}(g)\cap \mathcal{A}}w_{gj} }, \\ &\qquad \mathrm{for}\ l=0,\ldots,L_{\mathrm{str}}-1, \end{aligned}\end{align*}

where $\mathcal{N}(g)$ denotes the STRING neighbors of gene *g*, *λ * controls the self-feature contribution, and $L_{\mathrm{str}}=2$, *λ =0.5* are used by default. STRING edge weights are obtained by normalizing the retained STRING combined scores above 400 as $w_{gj}=(\mathrm{score}_{gj}-400)/(1000-400)$. The graph-smoothed vector $X_{g}^{\mathrm{string},(L_{\mathrm{str}})}$ is then partitioned into $K_{\mathrm{string}}$ groups, and each group is projected by a linear layer followed by layer normalization, GELU activation, and dropout:

(A.15)
\begin{align*}& z_{g}^{\mathrm{string}} = \mathrm{Tokenizer}_{\mathrm{string}} \left( X_{g}^{\mathrm{string},(L_{\mathrm{str}})} \right) \in \mathbb{R}^{K_{\mathrm{string}}\times d}.\end{align*}

The posterior ranker concatenates the view-specific tokens with a learnable classification token:

(A.16)
\begin{align*}& Z_{g} = \left[ z_{\mathrm{CLS}}; z_{g}^{\mathrm{gene}}; z_{g}^{\mathrm{brain}}; z_{g}^{\mathrm{string}} \right].\end{align*}

A learned type embedding $E_{\mathrm{type}}$ distinguishes the CLS, gene-level, BrainSpan, and STRING tokens. The Transformer input is

(A.17)
\begin{align*}& Z_{g}^{(0)} = Z_{g}+E_{\mathrm{type}}.\end{align*}

In the default implementation, the token dimension is *d=64*, the STRING view contains $K_{\mathrm{string}}=6$ tokens, and the sequence is processed by a two-layer Transformer encoder with four attention heads. Each layer applies multi-head self-attention followed by a position-wise feed-forward block with hidden dimension *\max (4d,64)=256*, GELU activation, dropout, and residual normalization:

(A.18)
\begin{align*}& Z_{g}^{(l+1)} = \mathrm{TransformerLayer}^{(l)} \left( Z_{g}^{(l)} \right), \qquad l=0,1.\end{align*}

The final Transformer output is $\widetilde{Z}_{g}=Z_{g}^{(2)}$. The CLS output is then layer-normalized to obtain the fused posterior multi-omics representation:

(A.19)
\begin{align*}& h_{g} = \mathrm{LN} \left( \widetilde{Z}_{g,\mathrm{CLS}} \right).\end{align*}

A shared bottleneck maps $h_{g}$ to a compact scoring representation:

(A.20)
\begin{align*}& b_{g} = \mathrm{Dropout} \left[ \mathrm{GELU} \left( W_{b} h_{g}+a_{b} \right) \right],\end{align*}

where the bottleneck dimension is *\max (d,64)*. Two task-specific heads are applied to $b_{g}$. The PU head produces the posterior PU score

(A.21)
\begin{align*}& s_{\mathrm{PU}}(g) = W_{\mathrm{PU}}b_{g}+a_{\mathrm{PU}},\end{align*}

and the ranking head produces the pairwise ranking score

(A.22)
\begin{align*}& r(g) = W_{\mathrm{rank}}b_{g}+a_{\mathrm{rank}}.\end{align*}

The score $s_{\mathrm{PU}}(g)$ enters the lower-bound PU objective $L_{\mathrm{LB\mathrm{-}PU}}$, whereas *r(g)* enters the lower-bound pairwise ranking objective $L_{\mathrm{LB\mathrm{-}rank}}$. Both losses use the entropy-shrunk negative responsibility $\widetilde{w}^{-}(g)$ defined above.

#### Multi-view posterior ranker and bound-aware objective

The bound-aware training objective contains the five losses shown in Fig. 2. First, the weighted lower-bound nnPU risk uses the entropy-shrunk negative responsibility $\widetilde{w}^{-}(g)$ for unlabeled genes:

(A.23)
\begin{align*} & L_{\mathrm{LB\mathrm{-}PU}} = \pi n_{P}^{-1} \sum_{g\in P_{L}} \mathrm{BCE}\!\left(s_{\mathrm{PU}}(g),1\right) + \left[ L_{\mathrm{neg}} \right]_{+},\end{align*}

(A.24)
\begin{align*} & L_{\mathrm{neg}} = n_{U}^{-1} \sum_{g\in U} \widetilde{w}^{-}(g) \mathrm{BCE}\!\left(s_{\mathrm{PU}}(g),0\right) - \pi n_{P}^{-1} \sum_{g\in P_{L}} \mathrm{BCE}\!\left(s_{\mathrm{PU}}(g),0\right). \end{align*}

where $L_{\mathrm{neg}}$ denotes the non-negative PU correction term and $[a]_{+}=\max (a,0)$. Second, the lower-bound pairwise PU ranking loss is

(A.25)
\begin{align*}& L_{\mathrm{LB\mathrm{-}rank}} = \frac{ \sum_{p\in P_{L}} \sum_{u\in U} \widetilde{w}^{-}(u) \log\left\{ 1+\exp[-(r(p)-r(u))] \right\} }{ n_{P}\sum_{u\in U}\widetilde{w}^{-}(u) }.\end{align*}

This weighting prevents unlabeled genes with low negative responsibility from being aggressively pushed below curated positives.

Third, the one-class positive compactness loss encourages embeddings of curated positives to remain close to the positive center:

(A.26)
\begin{align*}& L_{\mathrm{OC}} = \frac{1}{n_{P}} \sum_{g\in P_{L}} \left\| h_{g}-c_{P} \right\|_{2}^{2}, \qquad c_{P}= \frac{1}{n_{P}} \sum_{g\in P_{L}} h_{g}.\end{align*}

Fourth, graph consistency regularizes local STRING neighborhoods by aligning each unlabeled gene with its selected graph neighbors:

(A.27)
\begin{align*}& L_{\mathrm{graph}} = \frac{1}{|\mathcal{E}_{\mathrm{graph}}|} \sum_{(g,j)\in\mathcal{E}_{\mathrm{graph}}} \left\| h_{g}-\mathrm{stopgrad}(h_{j}) \right\|_{2}^{2}.\end{align*}

Here $\mathcal{E}_{\mathrm{graph}}$ denotes the fold-local neighbor pairs used during training. Fifth, augmentation consistency stabilizes PU predictions under independently masked and noise-perturbed views:

(A.28)
\begin{align*}& L_{\mathrm{cons}} = \frac{1}{n_{U}} \sum_{g\in U} \left[ \sigma(s_{\mathrm{PU}}(g)) - \sigma(s_{\mathrm{PU}}(\widetilde{g})) \right]^{2},\end{align*}

where $\widetilde{g}$ denotes an independently masked and noise-perturbed view of the same gene.

The complete Stage-II objective is given by

(A.29)
\begin{align*}& \begin{aligned} L_{\mathrm{Stage\mathrm{-}II}} &= \lambda_{\mathrm{PU}}L_{\mathrm{LB\mathrm{-}PU}} + \lambda_{\mathrm{rank}}L_{\mathrm{LB\mathrm{-}rank}} + \lambda_{\mathrm{OC}}L_{\mathrm{OC}} + \lambda_{\mathrm{graph}}L_{\mathrm{graph}}\\ &+ \lambda_{\mathrm{cons}}L_{\mathrm{cons}}. \end{aligned}\end{align*}

Unless otherwise specified, we use

\begin{align*} & (\lambda_{\mathrm{PU}},\lambda_{\mathrm{rank}},\lambda_{\mathrm{OC}}, \lambda_{\mathrm{graph}},\lambda_{\mathrm{cons}}) = (1.0,0.5,0.05,0.05,0.05), \end{align*}

which were selected as empirically validated default settings in the training experiments. This objective couples the latent-responsibility construction with posterior representation learning: the same conservative weight $\widetilde{w}^{-}(g)$ governs both the nnPU risk and the pairwise ranking penalty, whereas the compactness, graph-consistency, and augmentation-consistency terms regularize the posterior embedding space.

After Stage-II training, the final genome-wide score is obtained by reciprocal-rank fusion of the empirical prior and PU posterior evidence:

(A.30)
\begin{align*}& S_{\mathrm{final}}(g) = \lambda_{\mathrm{prior}} \frac{1}{k_{\mathrm{rrf}}+r_{\mathrm{prior}}(g)} + (1-\lambda_{\mathrm{prior}}) \frac{1}{k_{\mathrm{rrf}}+r_{\mathrm{PU}}(g)}.\end{align*}

Here, $r_{\mathrm{prior}}(g)$ and $r_{\mathrm{PU}}(g)$ denote the genome-wide descending ranks induced by $\hat{\pi }(g)$ and $s_{\mathrm{PU}}(g)$, respectively, and $\lambda _{\mathrm{prior}}\in [0,1]$ controls the contribution of the empirical-prior rank relative to the PU posterior rank.

#### Implementation details

For Deep-MOTIFs, the default configuration used *π =0.10*, *δ =0.60*, $w_{\min }=0.10$, $\lambda _{\mathrm{rank}}=0.50$, $\lambda _{\mathrm{prior}}=0.50$, and reciprocal-rank fusion constant $k_{\mathrm{rrf}}=60$; optimization used AdamW with learning rate $10^{-3}$, weight decay $10^{-4}$, batch size 256, a 10-epoch linear warm-up followed by cosine annealing, and early stopping on validation PR-AUC with patience 50. Gaussian noise augmentation with factor 3 and scale 0.4 was applied for training data augmentation.

All baseline hyper-parameters follow their respective publications. ForecASD was reproduced as a three-stage random-forest pipeline with 1000, 500, and 500 trees for the BrainSpan, STRING, and fusion branches. DeepND and DeepASDPred were trained as two-layer GCNs with hidden dimension 4 and dropout 0.5. FRN used 200 trees with $\sqrt{p}$-feature subsampling and class-balanced weighting selected by nested five-fold ROC-AUC grid search. Krishnan-SVM used a class-weighted linear SVM with *C=1.0*, squared-hinge loss, feature standardization, and isotonic-regression probability calibration. For the PU-learning baselines, nnPU-MLP used the non-negative PU risk estimator with class prior *π =0.03*, *β =0*, and *γ =1*. VPU-MLP used the variational PU objective with *λ =0.01*. Dist-PU-MLP used the label-distribution alignment objective with a 60-epoch warm-up, learning rates $5\times 10^{-4}$ and $5\times 10^{-5}$ before and after warm-up, entropy coefficients $2\times 10^{-3}$ and $4\times 10^{-3}$, Mixup parameter *α =6.0*, Mixup-entropy coefficient *0.04*, and Mixup loss weight 5.0. XGBoost used the default XGBClassifier setting with 100 boosting trees, maximum depth 6, and learning rate *η =0.3*. DeepGBM used a 300-tree XGBoost teacher with *η =0.05*, distilled into a 512D neural branch optimized with AdamW. TabNet used batch size 1024 and virtual batch size 128. SAINT used *d=32*, two layers, and four attention heads with self-supervised pretraining before fine-tuning. FT-Transformer used *d=64*, three layers, eight attention heads, and dropout 0.1. The complete training workflow is summarized in Algorithm A1.

#### Robust comparison with tuned tree-based baselines

We performed an additional tuning analysis for two strong tree-based baselines, XGBoost and DeepGBM, to assess the stability of the comparative results under alternative baseline configurations. For XGBoost, we varied the maximum tree depth from 3 to 7, which directly controls tree complexity and feature-interaction depth. For DeepGBM, we varied the GBDT distillation weight from 0 to 0.8, which controls the strength of teacher guidance from the gradient-boosted tree branch to the neural component. All other hyper-parameters were kept identical to those used in the main benchmark, and each configuration was evaluated with the same five-fold held-out protocol. As shown in Fig. A1, XGBoost achieved its best PR-AUC at maximum depth 4, whereas deeper trees did not yield additional improvement. DeepGBM showed a gradual increase in PR-AUC and ROC-AUC as the distillation weight increased, with the best ranking performance observed at 0.8. Nevertheless, the strongest tuned configurations of both baselines remained below Deep-MOTIFs across F1, PR-AUC, and ROC-AUC.
